# Current and future habitat suitability of northern fur seals and overlap with the commercial walleye pollock fishery in the eastern Bering Sea

**DOI:** 10.1186/s40462-025-00545-6

**Published:** 2025-04-14

**Authors:** Elizabeth A. McHuron, Elliott L. Hazen, Noel A. Pelland, Kelly A. Kearney, Wei Cheng, Albert J. Hermann, Rolf R. Ream, Jeremy T. Sterling

**Affiliations:** 1 Cooperative Institute for Climate, Ocean, and Ecosystem Studies, University of Washington, Seattle, WA USA; 2 Environmental Research Division, Southwest Fisheries Science Center, National Oceanic and Atmospheric Administration, Monterey, CA USA; 3 Institute of Marine Sciences, University of California, Santa Cruz, CA USA; 4 Alaska Fisheries Science Center, National Marine Fisheries Service, National Oceanic and Atmospheric Administration, Seattle, WA USA; 5 Ocean Environment Research Division, NOAA/Pacific Marine Environmental Laboratory, Seattle, WA USA; 6 Marine Mammal Laboratory, Alaska Fisheries Science Center, National Marine Fisheries Service, National Oceanic and Atmospheric Administration, Seattle, WA USA

**Keywords:** *Callorhinus ursinus*, Northern fur seal, Pribilof Islands, Regional ocean modeling, Species distribution models, Walleye pollock

## Abstract

**Background:**

Understanding the abiotic and biotic drivers of species distribution is critical for climate-informed ecosystem management. We aimed to understand habitat selection of northern fur seals in the eastern Bering Sea, a declining population that is also a key predator of walleye pollock, the target species for the largest U.S. commercial fishery.

**Methods:**

We developed species distribution models using random forest models by combining satellite telemetry data from lactating female fur seals tagged at different rookery complexes on the Pribilof Islands in the eastern Bering Sea with regional ocean model simulations. We explored how data aggregation at two spatial scales (Pribilof-wide and complex-specific) impacted model performance and predicted distributions. Spatial predictions under hindcasted (1992–2018) and projected (2050–2059) physical and biological conditions were used to identify areas of core habitat, overlap with commercial fishery catches, and potential changes in future habitat suitability.

**Results:**

The most important environmental predictor variables across all models were bathymetry, bottom temperature, and surface temperature. The Pribilof-wide model both under- and overrepresented the importance of specific areas, while complex-specific models exhibited considerable variability in transferability performance. The majority of core habitat occurred on the continental shelf in areas that overlapped with commercial catches of walleye pollock during the “B” season (June – October), with an average of 76% of the total percentage of the catch occurring in core fur seal habitat within the foraging range of lactating females. Projections revealed that considerable changes in fur seal habitat suitability may occur in the coming decades, with complex-specific variation in the magnitude and direction of changes.

**Conclusions:**

Our results illustrate the need to sample multiple sites whenever possible and consider spatial scale when extrapolating species distribution model output for central-place foragers, even when terrestrial sites are < 10 km apart. The high overlap between suitable fur seal habitat and commercial fishery catches of pollock, coupled with projected changes in habitat suitability, underscore the need for targeted studies investigating fisheries impacts on this declining population.

**Supplementary Information:**

The online version contains supplementary material available at 10.1186/s40462-025-00545-6.

## Background

Species distributions at fine- to meso-scales are influenced by local environmental conditions, either directly due to species’ physiological tolerances or indirectly through environmental impacts on food resources [1, 2]. Investigating the environmental factors that drive species distributions are crucial for species management, as it can allow for the identification of critical habitat, inform the delineation of protected areas, and aid in mitigation of interactions with human activities [3, 4]. Species Distribution Models (SDMs), statistical models that describe the relationships between species occurrence or abundance and abiotic and biotic variables or spatial characteristics [5], have thus become increasingly relevant as wildlife populations face rapidly changing environments that have already resulted in alterations to phenological timing, range shifts, and population dynamics [6–10]. In addition to facilitating climate-informed management of individual species [11, 12], SDMs provide insight into how future conditions might alter species interactions and impact socioeconomics of local communities [13, 14].

The Bering Sea is a high-latitude, semi-enclosed sea supporting a diverse biological community, valuable commercial fisheries, including walleye pollock (*Gadus chalcogrammus*, hereafter pollock), and Alaska Native communities [15]. This region has experienced reduced winter sea-ice coverage and increased water temperatures in recent years [16, 17], which has impacted species across multiple trophic levels [18–21] and altered predation landscapes [22]. Based on projected climate conditions [23, 24], single- and multi-species models predict steady declines of biomass in the next 80 years across most trophic groups in the eastern Bering Sea [25–28], with northward shifts in distribution for some invertebrate and fish species [12, 28, 29]. Forecasted biological changes may be mitigated, at least at short timescales, by ecosystem-based fisheries management [26], which aims to promote resilient marine ecosystems and fisheries by considering interactions across multiple trophic levels. Providing climate-informed assessments for key predators in the Bering Sea, like marine mammals that can have large trophic impacts [30] and often respond quickly to environmental change [31], can provide critical information on underrepresented components in ecosystem-based fisheries managemen

Northern fur seals (laaquda$\:\widehat{\text{x}}$ in Unangam Tunuu; *Callorhinus ursinus*) are a key pollock predator in the Bering Sea, with consumption estimates that rival that of predatory groundfish species [32]. In addition to their ecological role, northern fur seals provide food stability and are central to the cultural identity of Pribilof Island Unanga$\:\widehat{\text{x}}$ communities [33]. Fur seals breeding on the Pribilof Islands, which numbered in the millions in the 1950s, have experienced an ongoing decline since the mid-late 1990s of approximately 4% per year, primarily driven by declines at St. Paul Island [34]. Causes of the decline remain unresolved, but prey availability during reproduction is one hypothesized factor that also has contributed to a recent proposal to designate a national marine sanctuary around the Pribilof Islands [33]. While this fur seal population is one of the best-studied marine mammal populations worldwide, there is limited knowledge on how at-sea distributions are influenced by environmental conditions [35–39]. This data gap hinders the ability to make predictions about how projected environmental changes will impact fur seal population dynamics and cross-trophic and ecosystem impacts of such changes.

The purpose of this study was to predict habitat suitability of northern fur seals from the Pribilof Islands and identify how suitability is likely to change in the coming decades. Specific objectives included to (1) identify associations between fur seal presence and environmental conditions from telemetry data and regional ocean model simulations, and (2) use these relationships to predict recent (1992–2018) and future (2050–2059) habitat suitability. Our focus is on lactating females due to data availability and their importance in driving population dynamics [40]. Parental investment is provided solely by the female and as income breeders, female northern fur seals rely on resources in the eastern Bering Sea to support the costs of lactation. The results of this study contribute to ongoing efforts within the Alaska Climate Integrated Modeling Project [41] aimed at incorporating northern fur seals into ecosystem-based fisheries management of Bering Sea fisheries and providing advice for northern fur seal co-management efforts under the Marine Mammal Protection Act and by Pribilof Island Unanga$\:\widehat{\text{x}}$ communities.

## Methods

All analyses were conducted using R version 4.2.2 (R Core Team 2022). Spatial data were analyzed using a combination of the “sf” v. 1.0–16 [42, 43], “stars” v. 0.6-5 [42], “terra” v. 1.7-3 [44], and “raster” v. 3.6*–*26 [45] packages.

### Satellite telemetry data

**Fig. 1:**
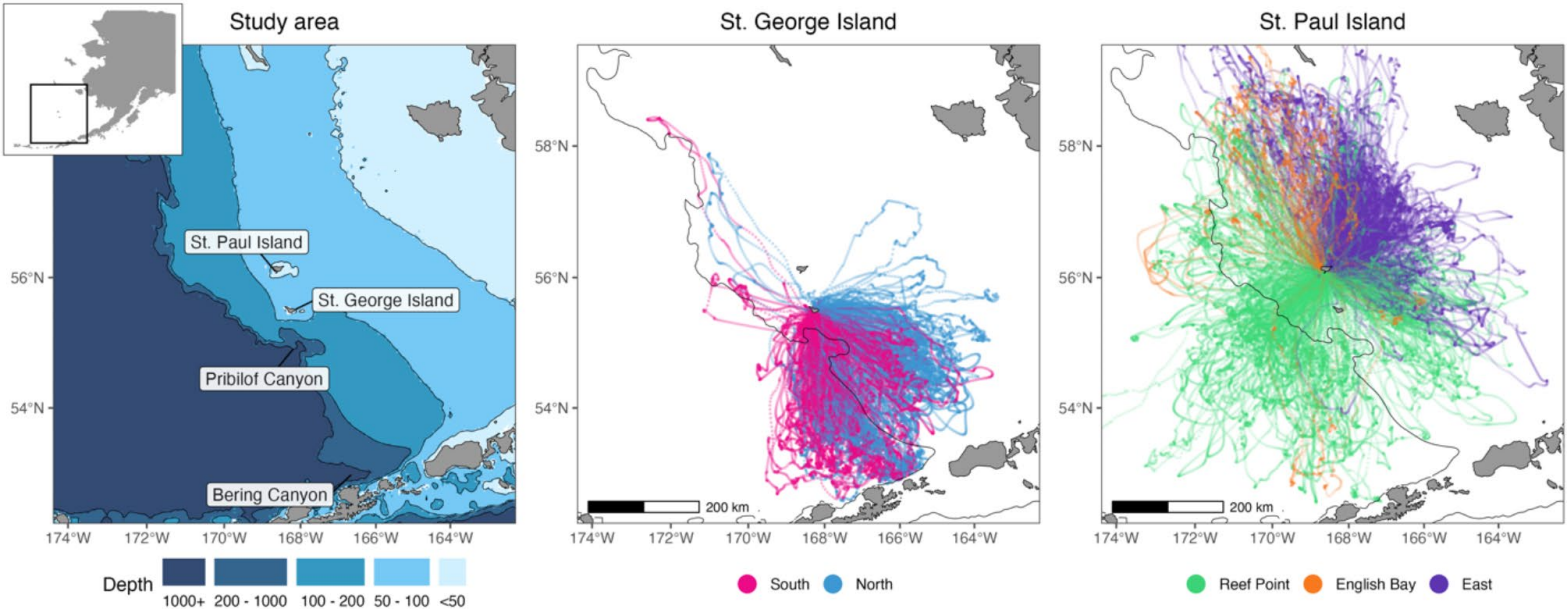
Study area and satellite telemetry data from lactating female northern fur seals on the Pribilof Islands. Inset plot shows location of study area in the Bering Sea. The locations of St. Paul and St. George Islands, part of the Pribilof Islands, are highlighted in addition to several notable physical features. Satellite tracks are colored based on rookery complex, separated by island. Only a subset of telemetry data are shown for the East complex on St. Paul Island. See Fig. S1 for the terrestrial locations associated with each complex

Lactating adult female northern fur seals from St. Paul (57.19º N, 170.25º W) and St. George Islands (56.60º N, 169.55º W) Alaska, USA were instrumented with satellite tags between 1992 and 2018 (Fig. 1). These are the two largest of the Pribilof Islands group, with St. Paul Island being the largest northern fur seal breeding rookery in U.S. waters. During the four-month lactation period (pups are born in July and wean in early November), adult females are central place foragers, alternating nursing visits at terrestrial sites with foraging trips to sea. Pups fast while females are at sea, remaining in the same general vicinity such that females depart from and return to the same terrestrial site. Instruments remained on fur seals for 1–14 foraging trips, with average (± standard deviation, SD) durations of 6.6 ± 2.4 days. Hourly locations were predicted using a continuous-time correlated random walk model (package “crawl” v. 2.3.0; [46, 47]). Trips were excluded if they were the start of the migration out of the Bering Sea. This resulted in data from 394 individual fur seals. Locations were obtained during all months of lactation, but 71% of these occurred during August and September, with lesser contributions from October (17%), July (8%), and November (4%). Additional details of instrumentation and processing can be found elsewhere [48–50].

### Environmental data

We downloaded environmental data from a hindcast simulation of a Regional Ocean Modeling System model for the Bering Sea via a publicly available server (https://data.pmel.noaa.gov/aclim/thredds/catalog/files/B10K-K20P19_CORECFS.html). Detailed documentation of the parameterization of this specific Bering Sea model domain and validation of its physical and biogeochemical variables under historical atmospheric and ocean forcing conditions are available elsewhere [51, 52]. In brief, the model domain, referred to in previous publications as Bering10K, simulates the Bering Sea region with a 10-km horizontal resolution and 30 terrain-following vertical layers. Biogeochemical processes are coupled to the ocean model via the Bering Ecosystem Study Nutrient Phytoplankton Zooplankton (BEST_NPZ) model, a Bering-focused biogeochemical model that simulates 2 size classes of pelagic phytoplankton; 5 size classes of zooplankton (microzooplankton, small and large copepods, euphausiids, and jellyfish); nitrogen, iron, and carbonate system dynamics; and simple sea ice and benthic biogeochemistry [51–53]. We hereafter refer to this Bering10K-BEST_NPZ ROMS model as ROMS-NPZ for brevity. The atmospheric and ocean lateral boundary forcings used to drive ROMS-NPZ are obtained from the NOAA Climate Forecast System Reanalysis and Climate Forecast System version 2 (CFSv2) Operational Analysis [54]. ROMS-NPZ state and diagnostic variables were archived at a weekly-averaged temporal resolution. For this study, the ROMS-NPZ output data were regridded from the native curvilinear coordinate grid to a regular longitude-latitude coordinate system with similar resolution using nearest neighbor resampling.

Environmental variables were selected based on previous studies on northern fur seals [35, 50, 55], their prey [56, 57], or other pinniped species [58–61], as well as preliminary investigations of variable collinearity among a suite of ROMS-NPZ physical and biological variables. Final variables included bathymetric depth (bottom depth relative to sea level, hereafter referred to as bathymetry), surface and bottom temperature, large phytoplankton biomass, and large copepod and euphausiid biomasses (Table 1). In addition to the large phytoplankton biomass value associated with the weekly ROMS-NPZ temporal archiving bin that occurred closest in time to a given fur seal location, we also calculated a value for each year by averaging large phytoplankton biomass values from April – July. The intent was that this average phytoplankton variable might better capture broader spatial patterns in phytoplankton biomass, particularly given temporal lags between primary productivity and upper trophic level responses. All biological variables were integrated over depth since fur seals may forage at depths > 100 m [62]. Northern fur seals are known to use mesoscale features such as eddies [35, 39], but no variables intended to capture these features were included because, while capable of generating mesoscale eddies with realistic spatiotemporal characteristics, the free-running ROMS-NPZ output without data assimilation is not expected to capture the exact location and timing of specific real-world eddies.

**Table 1:** Environmental variables used in northern fur seal habitat models, either directly output from the ROMS-NPZ model or calculated from model output

Variable	Units	Temporal resolution	Additional description
Bottom temperature	ºC	Weekly	Average temperature in the bottom 5 m
Surface temperature	ºC	Weekly	Average temperature in the top 5 m
Bathymetry	m	Static	Depth of ocean floor relative to sea level
Large phytoplankton (PhL)	mg C m^− 2^	Weekly	Biomass integrated over depth
Average large phytoplankton	mg C m^− 2^	Yearly	Average integrated biomass from April to July in each year
Large copepod (NCaO + NCaS)	mg C m^− 2^	Weekly	Biomass integrated over depth
Euphausiid (EupO + EupS)	mg C m^− 2^	Weekly	Biomass integrated over depth

The ROMS-NPZ variable name is shown in parentheses for select variables. Bathymetry was correlated with bottom temperature in the basin and was excluded from the analysis. Bottom temperature was retained because it is dynamic and has good skill. All other variables were included in both the basin and continental shelf models. Temporal resolution describes whether a given variable was static for a grid cell, or if dynamic, the timescale at which output was aggregated

### Pseudo-absences

Since satellite telemetry data are presence-only, it is necessary to generate locations for where seals could have gone but did not (pseudo-absences). Pseudo-absences were generated using the approach of [63]. We fit a first-order vector-autoregressive model to each trip (package “availability” v. 0.15.0; [64]) and simulated 100 tracks that resembled the original track with respect to speed and duration (Fig. S2). Because northern fur seals are central place foragers during the breeding season, we used fixed starting and ending points (the rookery) as in the original trip. Pseudo-absences were filtered so none occurred within the same or adjacent ROMS-NPZ grid cells as any of the presence data within a six-day period [65]. We chose a six-day interval because ROMS-NPZ simulations were weekly-binned averages.

### Combining datasets

Environmental data were extracted at each fur seal presence and pseudo-absence, using nearest neighbor interpolation when fur seal locations did not occur on the same day as ROMS output. Locations were assigned to a habitat (continental shelf, basin) based on the ROMS-NPZ bathymetry (shelf ≤ 200 m, basin > 200 m). Most trips consisted of locations that were exclusively (84%) or predominately (14%) in one habitat type, with only 2% of trips that were more equal in their distribution of locations between basin and shelf habitat (< 75% of locations in one habitat type). Because the ROMS-NPZ continental shelf is narrower than observations due to necessary bathymetric smoothing [51], we removed locations that occurred within areas where the ROMS-NPZ depth was inconsistent with observations (i.e., ROMS-NPZ bathymetry > 200 m but real-world location on the shelf). We also removed locations within the first and last 12 h of a trip because these locations are primarily associated with transit to and from the rookery.

Locations (presence and pseudo-absence) were randomly down-sampled to a single location per day to minimize temporal autocorrelation. We paired each trip with a simulated trip to create a dataset with a 1:1 presence to absence ratio. To select the simulated trip, we ranked the 100 simulated trips based on the total number of daily locations and selected the trip with the highest number of days to maximize sample sizes. Additional locations were dropped (either presence or pseudo-absence) when there was a temporal mismatch between the two trips (i.e., a location was not present on a given day for both the simulated and actual trip), which sometimes occurred if a trip contained both shelf and basin locations. We repeated this process ten times for each habitat to incorporate and assess how uncertainty in daily locations affected model output. For the actual trip, this was done by randomly selecting different daily locations, while for the simulated trip this was accomplished by selecting a different trip. While each replicated dataset consisted of the same number of presence and pseudo-absences, there were slight differences in sample sizes among replicate datasets.

### Habitat modeling

We used random forest (RF) classification models to assess correlative relationships between fur seal presence/absence and environmental variables for each habitat type, with separate models fit to each replicate dataset. Random forest is a supervised machine learning algorithm that builds ensembles of decision trees, where individual trees are generated from random subsets of features and input data. We chose RF over other common modeling approaches where presence data are derived from satellite telemetry (e.g., generalized additive models, boosted regression trees) because of its fast implementation, minimal assumptions, and relative insensitivity to model input parameters. Preliminary exploration that included boosted regression trees also resulted in broadly similar results as RF models. Prior to analysis, variable correlations were assessed for each habitat type. Bathymetry and bottom temperature were strongly correlated (*r >* 0.7) in the basin; we chose to include bottom temperature instead of bathymetry since it is a dynamic variable.

We fit RF models using the *ranger* function (package “ranger” v. 0.16.0; [66]) implemented via the *train* function (package “caret” v. 6.0–93; [67]), which selects the optimal input parameters and fits the final model to the full dataset using optimally selected parameters. The optimal number of variables to randomly sample as candidates at each split (we explored values of 2, 3, or 4) was selected using 10-fold cross validation (CV) for each replicate dataset, with folds grouped by trip. We did not explore any values higher than four because models typically perform well under default settings (in our models the default value was two) and to minimize computation time given the number of models that were run [68]. The area under the receiver operating characteristic curve (AUC) was used as the performance metric to identify optimal parameter values. Default settings were used for all other model parameters, such as the number of trees (500) and minimum node size (1). Since we had a priori knowledge that lactating female northern fur seals use space differently depending on the location of rookeries [69], we ran models that included all data from a given habitat type (referred to as the Pribilof model) as well as models on data subset based on spatial patterns of habitat use. We used designations from [70] to subset datasets, who identified five distinct rookery complexes (collections of rookeries in geographical proximity) based on clustering of diet data, three on St. Paul Island (East, English Bay, and Reef Point) and two on St. George Island (North and South; Fig. S1). This resulted in a total of 11 model combinations, each with 10 replicate datasets. This was one less than the total number of potential combinations because, as very few fur seals from East use the basin, there was no basin model for this complex.

Model performance was assessed based on AUC values from the 10-fold CV of the best model (AUC_CV_ in Table 2) and the exploration of spatial and temporal performance. The inclusion of spatial and temporal performance provided an indication of how well individual models performed when they were used to predict occurrence for complexes (spatial) or years (temporal) not used to fit the model. For the Pribilof models, performance of spatial (by complex, AUC_Space_ in Table 2) and temporal folds (by year, AUC_Time_ in Table 2) was assessed using the *train* function as described above. Folds did not necessarily contain a single complex or year, as the function used to create folds attempts to balance sample sizes across folds. While we included temporal folds, they are somewhat confounded by space because a single year may only be represented by one complex due to sampling design. For the complex-specific models, temporal folds were assessed in the same way as for the Pribilof models, however, spatial performance was based on the AUC of model predictions to other complexes (i.e., model transferability). For all performance metrics, we used the *evalm* function (package “MLeval” v. 0.3; [71] to calculate AUC values. AUC values range from 0 to 1; values of 0.5 (or less) indicate a model is no better than random, 0.7 and greater are generally indicative of a model that has good (or better than good) discriminatory power, and a value of 1 indicates perfect discrimination. Values for several other performance metrics output by *evalm* associated with the 10-fold CV are provided in Table S1, including sensitivity and specificity.

**Table 2:** Model sample sizes and performance metrics for northern fur seal habitat selection models

Model	*n*_seal_	*n*_trip_	*n*_year_	AUC_CV_	AUC_Time_	AUC_Space_
*Continental shelf*						
Pribilof	331	1,272	21	0.88 ± 0.005	0.62 ± 0.02	0.51 ± 0.04
East	176	868	16	0.91 ± 0.004	0.76 ± 0.001	0.23–0.58
English Bay	12	39	3	0.86 ± 0.03	0.77 ± 0.07	0.44–0.71
Reef Point	65	199	12	0.74 ± 0.02	0.62 ± 0.02	0.51–0.79
North	49	123	7	0.95 ± 0.01	0.87 ± 0.02	0.28–0.97
South	18	43	6	0.96 ± 0.01	0.80 ± 0.07	0.25–0.91
*Basin*						
Pribilof	148	363	16	0.81 ± 0.006	0.67 ± 0.02	0.71 ± 0.02
English Bay	5	10	3	0.74 ± 0.07	-	0.46–0.58
Reef Point	65	167	12	0.66 ± 0.02	0.53 ± 0.04	0.53–0.69
North	36	65	7	0.89 ± 0.01	0.83 ± 0.02	0.53–0.93
South	40	119	6	0.94 ± 0.01	0.86 ± 0.03	0.55–0.91

Models for each habitat type were run using all of the data (Pribilof models) as well as on subsets based on rookery complex. Sample sizes represent the number of fur seals (n_seal_), number of foraging trips (n_trip_), and number of years (n_year_) within each model, averaged across the 10 replicate datasets with the associated standard deviation. Mean performance metrics (replicate average ± SD), evaluated using the area under the receiver operating curve (AUC), are shown for 10-fold cross-validation (AUC_CV_), as well as temporal (AUC_Time_) or spatial blocking (AUC_Space_; Pribilof models only). Values for AUC_Space_ for complex-specific models represent the mean model performance in predicting to datasets from other complexes, presented as a range across complexes

Variable importance was assessed using the *varImp* function (package “caret”) and the Gini importance measure, which is calculated by summing the weighted impurity decreases for all nodes associated with a given variable and then averaging across all trees (*importance* function in “ranger”). Importance was scaled (the most important variable received a value of 100 and the least important variable a value of 0) and then averaged across all replicate models. Partial dependence plots were created using “DALEX” v. 2.4.3 [72], which illustrate the relationships between model predictions and individual environmental variables (marginal effects).

### Spatial predictions - hindcast

We generated spatial predictions of habitat suitability under hindcasted conditions (1992–2018), where more suitable habitat was assumed to occur in areas with a higher probability of occurrence, and less suitable under lower probability of occurrence. Each replicate model was used to predict the probability of occurrence per weekly ROMS-NPZ output between July and October in each year, which were then averaged to create a single weekly predictive surface that incorporated uncertainty in the specific presence-absence locations for each model type. Within the area of bathymetric mismatch, predictions were averaged between the shelf and basin models. We averaged the single weekly predictive surfaces across (1) the entire hindcast, and (2) during “warm” (1993, 1998, 2001–2005, 2014–2016, 2018) and “cold” (1992, 1994–1995, 1997, 1999, 2007–2010, 2012–2013) years. Year categories were based on designations from other papers, which are primarily based on summer water temperatures on the eastern Bering Sea shelf [73, 74]. All other years were assumed to represent “average” conditions. To limit predictions to areas that could theoretically be accessed by females from each island, the spatial extent was cropped to a 375 km radius circle centered between St. Paul and St. George Islands. This value was based on the maximum straight-line distance satellite-tagged lactating fur seals traveled from the rookery, which indicated that the 95% quantile of all trips was 350 km. We increased the value to 375 km to account for the centered position of the circle between the two islands. Core habitat was identified as cells with a probability of occurrence within the top 25% quantile from the averaged predictions across the entire hindcast; separate core habitat thresholds were identified for each model. Thresholds for cells within the area of bathymetric mismatch were based on the average of the basin and shelf thresholds.

To provide a preliminary assessment of overlap between fur seals and the commercial pollock fishery, we overlaid fishery catches from the pollock “B” season (catches from June – October) on predictions of core habitat. Pollock catches, as estimated by scientifically trained observers, were compiled and averaged at a spatial resolution of 0.25° x 0.25° latitude and longitude over 2000–2022 (J. Ianelli pers. comm). This provided the relative footprint of pollock fishery removals; it is not absolute in part because not all tows have good spatial references, and because our dataset did not include October catches. We did not use data prior to 2000 because of lower coverage for estimating spatially explicit catches. Overlap was visualized using an additive color mixing approach. Cells were identified as either being core fur seal habitat for at least one complex (a value of one) or not containing core habitat for any complex (a value of zero). Core habitat was resampled to the coarser spatial grid of the commercial fisheries data by averaging values from all fur seal cells that occurred partially or completely within a pollock grid cell. Fur seal core habitat values and pollock catches were each plotted using gradient color scales of white – blue (fur seal) or white – red (fishery), where white represented the absence of core habitat or fishery catches. Colors were extracted and additively mixed to identify areas of overlap (purple) using “colorspace” v. 2.1-1 [75]. The average proportion of pollock extracted by the fishery within core fur seal habitat was calculated by summing the mean proportion of the total catch within each 0.25° x 0.25° grid cell with a core habitat value > 0. A more comprehensive assessment of overlap between fur seals with the commercial pollock fishery, or other analyses investigating the effect of fishery activities on fur seal distribution, was outside the scope of this study.

### Spatial predictions - projections

In addition to hindcasts, we generated spatial predictions of average habitat suitability under projected (July – October from 2050 to 2059) conditions. The projections used the same ROMS-NPZ model configuration as the hindcast simulation but with future climate forcing conditions provided by the Coupled Model Intercomparison Project Phase 6 (CMIP6) archive, representing two different shared socioeconomic pathways (SSP126 - sustainability, SSP585 - fossil-fueled development) from three global Earth System Models (GFDL-ESM4, MIROC-ES2L, CESM2). The ROMS-NPZ variables from these projections were first bias-corrected to account for any systematic offsets in each Earth System Model. Weekly climatological bias offsets were calculated by comparing the historical portion of each downscaled forecast simulation (1985–2014) to the corresponding period from the reanalysis-forced hindcast simulation and then applied to the entirety of each time series. See Additional Material 1 for detailed methods related to this bias correction. Average projected habitat suitability was calculated in the same way as the hindcast, first by creating a single weekly average surface and then by averaging all predictions across the entire projection period.

We used three metrics to assess changes in habitat suitability between hindcasted and projected conditions, focusing just on core habitat: mean suitability, distance from the rookery, and the total area of core habitat. Metrics were calculated separately for basin and shelf models. The thresholds identified from the hindcasted predictions were applied to identify core habitat in projections. The distance between the rookery and core habitat was calculated by averaging the latitude and longitude of cell centroids, weighted by the probability of occurrence (the center of gravity). Rookery coordinates were approximated from those of St. Paul (East, English Bay, Reef Point) or St. George (North, South).

Extrapolation occurs when model predictions are made outside of the range of the values used to fit the model, which is a potential concern when projecting habitat suitability under rapidly changing or anomalous conditions. We assessed the extent of extrapolation for projected environments using “dsmextra” v.1.1.5 [76], an approach proposed by [77] that provides information on whether projected environmental conditions are (1) within the range of individual variables in the reference sample (analogue), (2) outside the range of individual variables in the reference sample (univariate), or (3) novel combinations of values within the univariate range of reference variables (combinatorial). It also identifies which variables have the largest contribution to extrapolation for any given grid cell and the spatial variation of extrapolation. We calculated weekly extrapolation values for each Earth System Model-SSP combination using the July – October hindcast simulations from 1992 to 2018 as the reference values.

## Results

The number of daily locations (presence or absence) included in the Pribilof models ranged from 4,876 to 5,517 (shelf) and 1,533–1,611 (basin), with a maximum of 339 and 152 individuals contributing to each analysis, respectively. The datasets were unbalanced with respect to the contribution of different complexes (Table 2), due not only to variation in sampling effort but also the specific behaviors of females from each complex (e.g., the length of trip durations, the utilization of different habitats). In particular, the English Bay complex was underrepresented in both habitats due to limited tracking data, and the East complex was overrepresented in the shelf model.

**Fig. 2:**
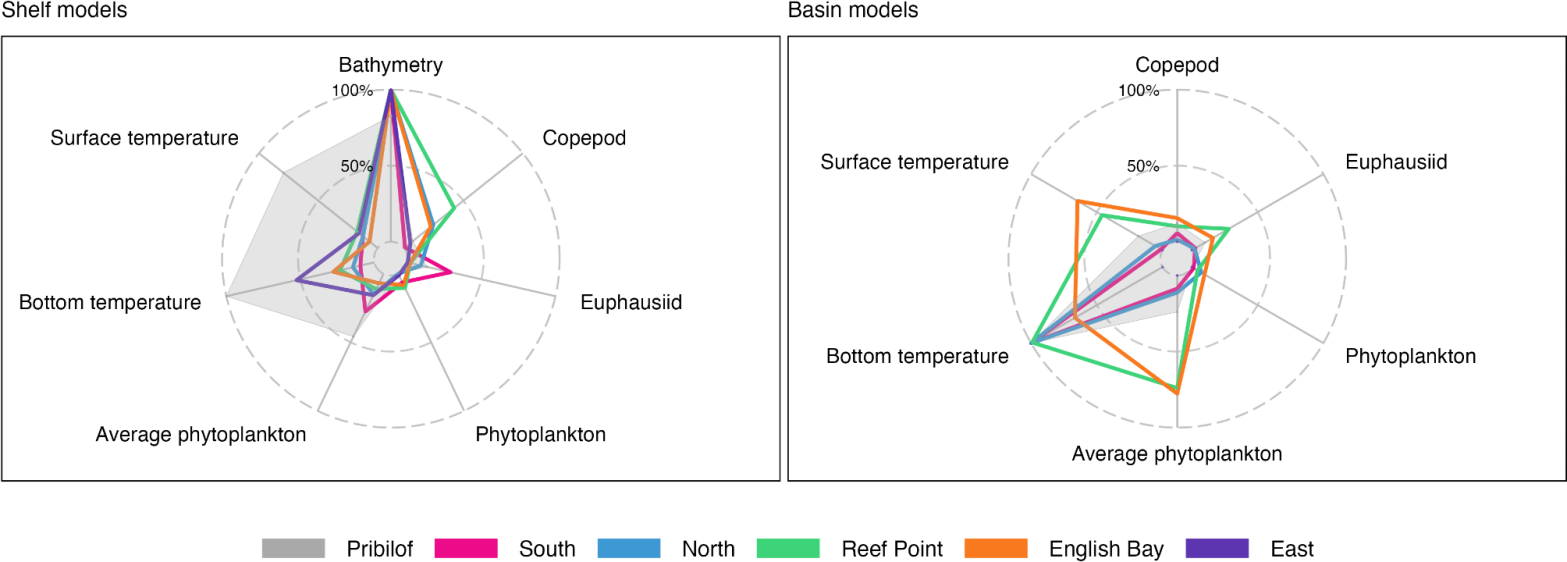
Relative importance of model variables in explaining continental shelf and basin habitat selection models. Results are shown separately for the Pribilof models (gray shaded area) and the complex-specific models (colored lines)

**Fig. 3:**
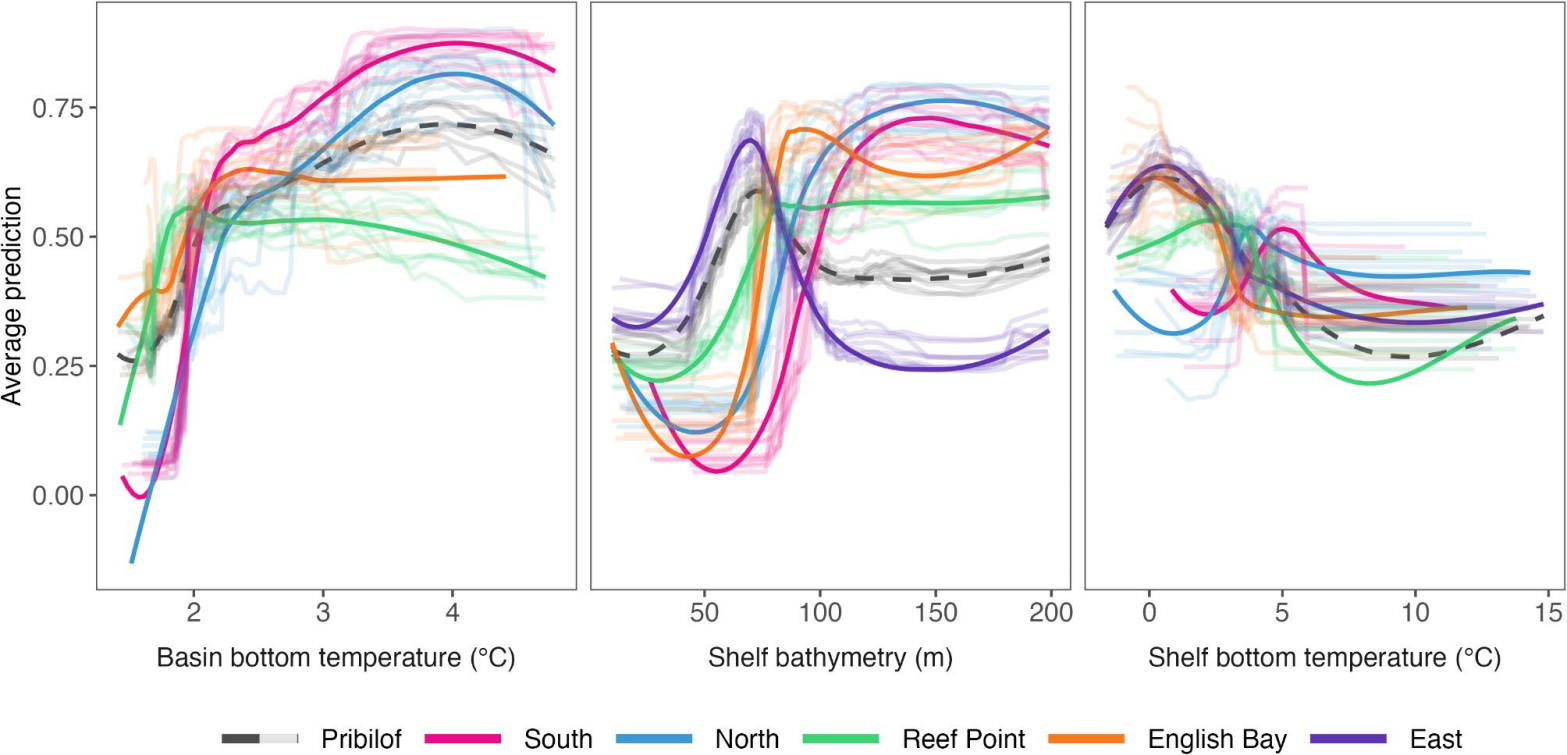
Partial dependence plots for bottom temperature and bathymetry from habitat selection models. Dashed black lines represent the Pribilof models, while colors depict complex-specific models. Individual lines represent the predictions from each replicate dataset. The bold line is a loess smoother intended to highlight the general relationship observed across all replicate datasets for a given model. Results are shown separately for the basin and shelf models, as indicated on the x axis

The most important environmental predictor variables across all analyses were bathymetry, bottom temperature, and surface temperature (Fig. 2). There were some differences in the relative importance of specific variables between the Pribilof and complex-specific models, as well as between individual complex-specific models. For example, bottom temperature was the most important variable in the Pribilof shelf model and the second most important variable for two of the three complex-specific models on St. Paul Island (East and English Bay), but was less important in St. George shelf models (Fig. 2). While bottom temperature was important across all basin models, several additional variables were important for the English Bay and Reef Point complexes (Fig. 2). Even when the importance of a predictor variable was shared across models, the specific relationship to the predicted occurrence was not always the same (Fig. 3, Figs. S3-S9). For example, females from East selected for shelf habitat with bathymetry between 50 and 100 m and against habitat with depths > 100 m. In contrast, females from all other complexes tended to select for habitats from 75 to 200 m in depth (Fig. 3). Similarly, while all shelf models indicated selection against bottom temperatures > 5–6 °C, females from English Bay and East also selected for cooler bottom temperatures (Fig. 3).

Model performances based on 10-fold CV were generally good, with AUC values for most continental shelf and basin models > 0.8 (Table 2). The Reef Point models performed the poorest compared with other complex-specific models, with mean AUC values of 0.74 (shelf) and 0.66 (basin). In general, model performance was slightly better for a given shelf model compared with its basin counterpart. Performance in predicting habitat use of specific years was still good for most models, although reduced compared with AUC values from 10-fold CV. There was no consistent pattern in the performance of cold vs. warm years across models, except that the mean CV of folds that contained warm years tended to be higher (AUC = 0.8) for the East shelf models than those that contained cold (AUC = 0.67) or average years (AUC = 0.71). Complex-specific model performance in predicting habitat use from other complexes was highly variable. All complexes performed poorly in predicting habitat use of at least one other complex, with minimum mean AUC values of 0.23–0.55 across basin and shelf models (Table 2). The East complex model performed poorly for all other complexes (AUC = 0.23–0.58), which was the driving factor behind the poor spatial performance of the Pribilof shelf model. Models from the two complexes on St. George Island exhibited good transferability to each other in both habitats (AUC > 0.9), but the same did not necessarily hold for St. Paul Island complexes.

**Fig. 4:**
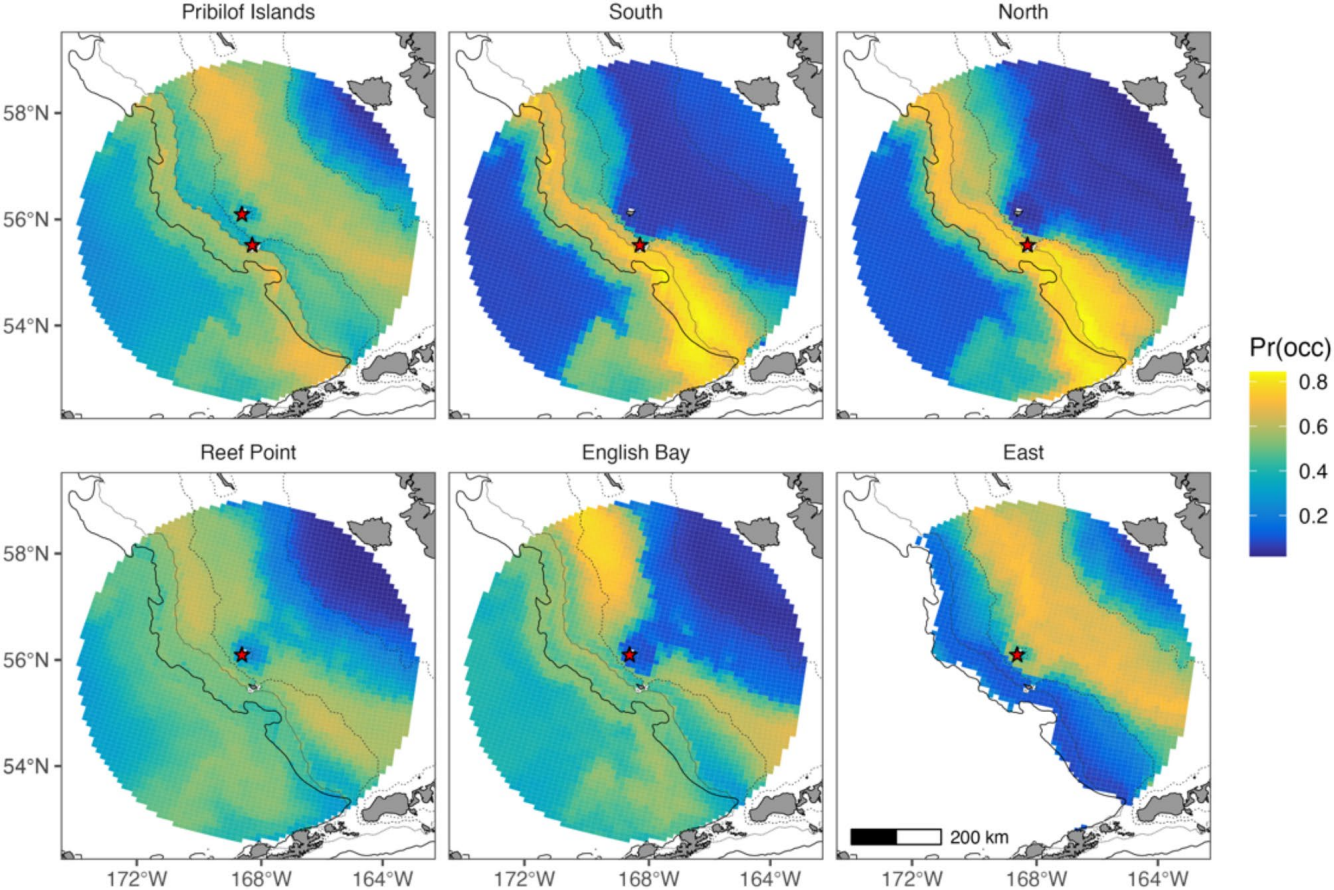
Spatial predictions of mean habitat suitability under hindcast conditions (1992–2018). Predictions are shown separately for each model. Red stars denote the island where each complex is located (either St. George Island or St. Paul Island, collectively the Pribilof Islands). The solid black line represents the 200 m isobath (from ETOPO 2022), whereas the dotted lines represent the ROMS-NPZ 200 m, 100 m, and 50 m isobaths (from left to right). Predictions between the actual and ROMZ-NPZ 200 m isobath represent average predictions from shelf and basin models. The scale bar is the same across all subplots

**Fig. 5:**
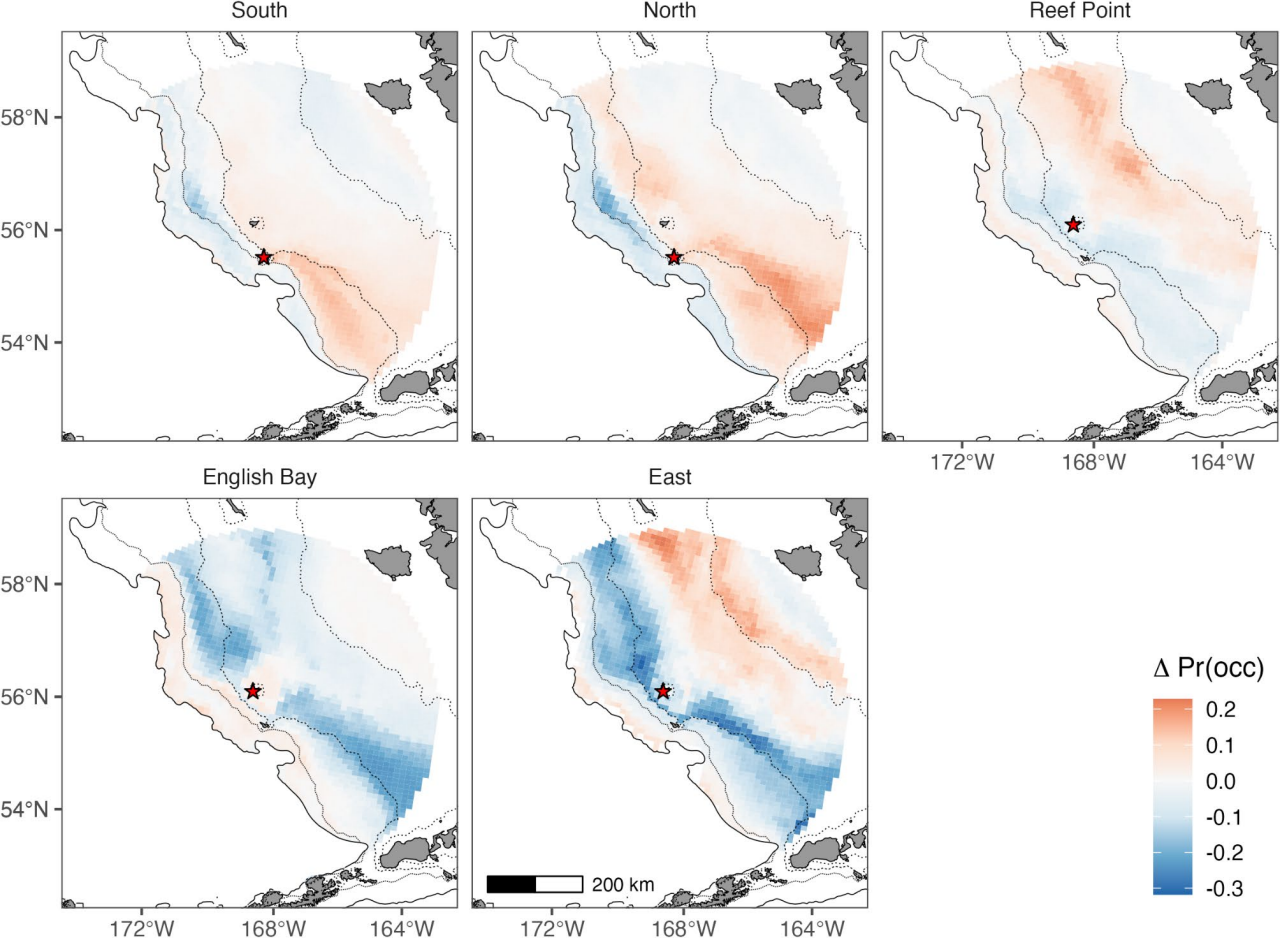
Spatial differences in mean habitat suitability between “warm” vs. “cold” years under hindcast conditions (1992–2018). Predictions are shown separately for each complex-specific model and restricted to the continental shelf. Positive values (red) correspond to areas that had higher mean habitat suitability during warm years, while negative values (blue) corresponded to areas that had higher mean habitat suitability during cold years. Red stars denote the island where each complex is located (either St. George Island or St. Paul Island). The solid black line represents the 200 m isobath (from ETOPO 2022), whereas the dotted lines represent the ROMS-NPZ 200 m, 100 m, and 50 m isobaths (from left to right). The scale bar is the same across all subplots

Complex-specific variation in habitat selection influenced the spatial predictions of habitat suitability (Fig. 4). Because of this variation, the Pribilof models under- and overrepresented the importance of specific areas for a given complex. For example, the area to the SE of St. George Island between the 100 and 200 m isobath was not of particular importance in the Pribilof model despite it being highly suitable habitat for both North and South complexes, likely due to the strong bias of the East complex in the shelf dataset. Similarly, basin habitat along the continental slope near the Bering Canyon was highly suitable habitat for fur seals from the North and South complexes but was of much less importance to fur seals from Reef Point and English Bay that tended to use basin habitat to the south and southwest of the Pribilof Islands. Comparisons between warm and cold years revealed some spatial changes in habitat suitability, particularly at East where habitat suitability increased in the inner middle and inner shelf during warm years (Fig. 5).

**Fig. 6:**
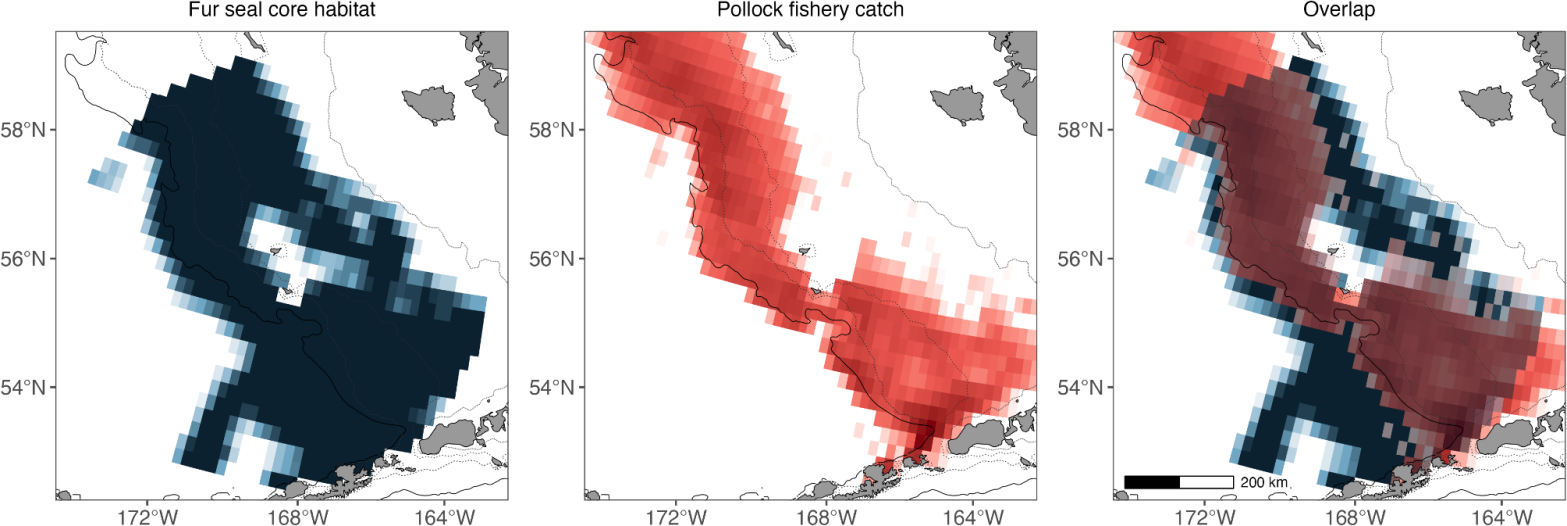
Overlap between fur seal core habitat and commercial fishery catches of walleye pollock. Colors indicate areas of core fur seal habitat (blue), fishery catches (red), and overlap between the two (purple), with deeper colors corresponding to higher values of core habitat (range of 0–1) or fishery catches (range of 0–27,797 t on a log scale). The entire spatial extent of fishery catches is not shown. The solid black line represents the 200 m isobath (from ETOPO 2022), whereas the dotted lines represent the ROMS-NPZ 200 m, 100 m, and 50 m isobaths (from left to right). The scale bar is the same across all subplots

Core fur seal habitat included continental shelf habitat to the northwest, southeast, and west of the Pribilof Islands, shelf break, and basin (slope and into the basin) habitat to the south and southeast of both islands (Fig. 6). Habitat around each of the islands was generally not identified as core habitat, although it is used by fur seals as they depart from and return to terrestrial rookeries. The total area of all cells identified as core habitat for at least one complex within the 375 km circle was 258,078 km^2^, with 67% of the total area of core habitat occurring on the continental shelf. The majority of fur seal core habitat overlapped with commercial pollock fishery catches (60% by area), with unfished areas of core habitat occurring in the basin and along a part of the middle shelf (Fig. 6). When just considering continental shelf habitat, an average of 77% of the available area of core habitat occurred in fished areas. The average proportion of the total pollock catch that occurred within core fur seal habitat (in the 375 km circle) was 76%.

Projected conditions from 2050 to 2059 indicated a general warming trend in both surface and bottom waters, with the greatest warming occurring under the CESM model (Figs. S10-S11). Most projected conditions were within the range of what was observed in the hindcasted years, with some instances of univariate extrapolation, primarily for large phytoplankton, copepod, and euphausiid biomass (Figs. S12 - S13). In all instances associated with these three biological variables, extrapolation was due to zeroes or values < 1, indicating these results may be an artifact of bias correction and are unlikely to influence habitat suitability predictions given the absolute range of biomasses. Excluding these variables, there were very little of the projection interval with extrapolated conditions across all Earth System Model-SSP combinations (Fig. S12), with most instances occurring along the outer extent of the foraging range on the inner shelf.

**Fig. 7:**
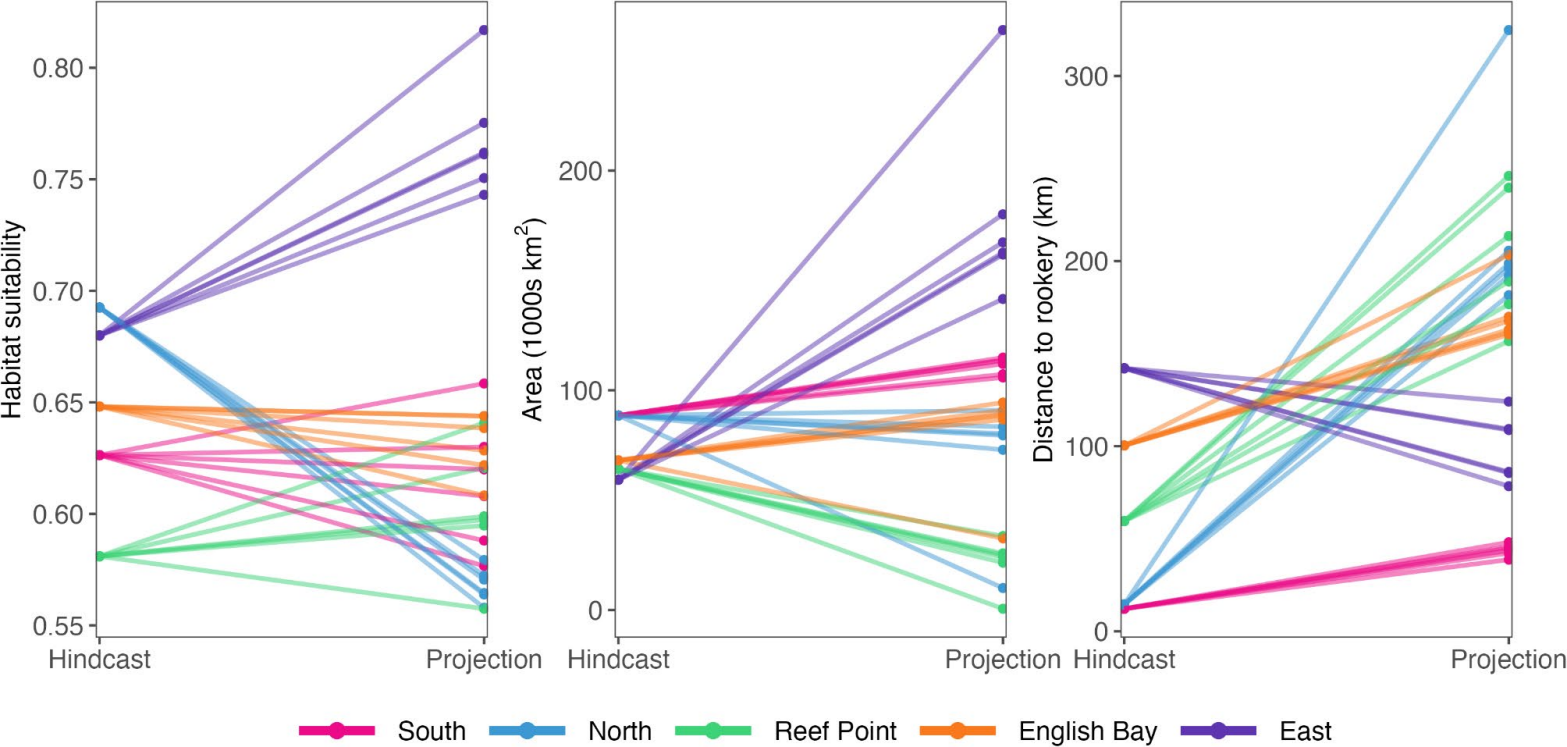
Changes in fur seal habitat suitability metrics on the continental shelf between hindcasts (1992–2018) and projections (2050–2059). Metrics shown are the mean core habitat suitability, the total area of core habitat, and the mean distance between the center of gravity of core habitat and either St. Paul or St. George Island. Each projection point represents a single Earth System Model-shared socioeconomic pathway combination

Variation in projected habitat suitability was greater among Earth System Models than SSP scenarios (Figs. S14 - S18), although most Earth System Model-SSP combinations projected similar changes in the direction of core habitat suitability metrics within a complex (Fig. 7). The magnitude and direction of changes in core habitat suitability metrics between hindcasted and projected conditions differed considerably among complexes and habitats (Fig. 7, S19). For example, at the North complex, the mean suitability of core habitat on the continental shelf was projected to decrease by 20–25%, with the center of gravity shifting considerably further from the island (Fig. 7, S14). In contrast, large gains in core habitat area and overall suitability were predicted for the East complex, particularly under the CESM model, with the core habitat shifting closer to the island (Fig. 7, S16). These projected gains appeared to be driven by changes in surface temperatures (i.e., weak selection for cold temperatures ca. 5 °C and stronger selection for warm temperatures ca. 10–13 °C), that may result from seasonal water column stratification on the middle shelf.

## Discussion

We combined satellite telemetry datasets collected from 394 lactating females over two decades with a regional ocean model simulation to identify habitat selection and suitability of northern fur seals in the Bering Sea. Identifying areas of suitable habitat is a useful management tool for northern fur seals, and valuable input for analyses quantifying spatial overlap with prey populations and anthropogenic activities that can inform ecosystem-based fisheries management. Projections of habitat suitability are also an important tool for understanding how changing conditions in the Bering Sea might affect northern fur seals and alter trophic interactions with key prey species like pollock that are also expected to be impacted by such changes [26, 78].

### Pribilof Island vs. complex-specific models

Central-place marine foragers often exhibit partial or complete spatial segregation in foraging areas associated with terrestrial resting and breeding sites [79–82]. The underlying mechanisms that may contribute to the emergence and maintenance of spatial segregation include the reduction of intraspecific competition, proximity of terrestrial sites to productive foraging regions, and memory [82–84]. For female northern fur seals, site-specific spatial segregation in foraging areas and diet have been well documented [48, 50, 69, 70, 85], although the underlying causes are not well understood. While it may not necessarily give rise to differences in habitat selection, several studies have found that model performance can be adversely affected when pooling data across colonies or regions [86, 87]. Model transferability may also be adversely affected, requiring more spatially and temporally extensive datasets to adequately characterize habitat suitability [88, 89]. While the Pribilof models performed well based on the 10-fold CV, part of this was likely an artifact of sample composition, and spatial predictions failed to highlight important habitat for specific complexes (referred to as homogenization by [90]). These issues were most apparent for the shelf model, which was not only driven by the strong bias towards the East complex on St. Paul Island, but also because of opposing relationships that minimized or neutralized the effect of important predictor variables. Our findings underscore the need to consider spatial scale during habitat model development and exercise caution in extrapolating models derived from one area to another, even at fine spatial scales where terrestrial sites are < 10 km apart. This is particularly true when extrapolating across areas with strong environmental gradients, as is the case for the eastern Bering Sea shelf.

### Habitat selection

Bathymetry and bottom temperature were both important in influencing habitat selection of northern fur seals in continental shelf habitat. Fur seals selected for habitat within the middle and outer shelf in waters with bottom temperatures < 6 °C, which is broadly consistent with the depth and temperature ranges of prey species consumed on the shelf, primarily pollock but also Pacific herring (*Clupea pallasii*), sablefish (*Anoplopoma fimbria*), and Atka mackerel (*Pleurogrammus monopterygius*; [56, 57, 91, 92]). Selection against inner shelf habitat (< 50 m) is likely a combination of reduced productivity and prey in this domain coupled with the fact that fur seals must travel further from rookeries to reach inner shelf habitat. Complex-specific differences in bottom temperature associations reflect differential use of the middle shelf domain, where bottom temperature is cooler because of a pool of cool summer bottom water (< 2 °C) that arises due to winter sea ice dynamics (referred to as the “cold pool”; [93]). The influence of other predictor variables was more complex-specific, with relationships that were generally more variable depending on the specific presences and pseudo-absences in the model. Several biological variables, such as large phytoplankton and euphausiid biomass, did influence habitat selection, although many of these relationships appeared to be solely driven by selection against areas with very low biomass. In addition, many of the ROMS-NPZ biological variables have reduced or unquantified skill [51], complicating interpretation of relationships. We included them under the assumption that they might still capture important processes or features relevant to fur seals, which appears to be the case for at least the South complex where outer shelf habitat near the Bering Canyon had elevated euphausiid and phytoplankton biomass.

Performance metrics indicate that our models successfully captured the broad-scale patterns of fur seal habitat suitability on the continental shelf. They were slightly less successful at capturing the nuances of interannual variation in distribution, which is not entirely unexpected given that other factors that influence spatial distribution are likely to be important at this temporal scale, such as the specific identity of tracked individuals. Like many otariids, northern fur seals exhibit consistent individual differences in foraging site fidelity [48], particularly at Reef Point where performance metrics were the poorest. Despite this, spatial predictions of differences in shelf habitat suitability between warm and cold years at St. Paul complexes were generally consistent with known responses of key prey species, namely pollock. Pollock tend to be more widely distributed across the shelf and shifted northwards during warm years, whereas distributions during cold years are more confined to the outer shelf (outside of the cold pool) or occur along the middle shelf domain within the cold pool itself (juveniles only; [2, 20, 57, 74, 94, 95]). The most pronounced changes were predicted for the East complex, which is consistent with their strong association with the middle shelf domain where the cold pool occurs. Fur seals from the North and South complexes exhibited different patterns of spatial changes in habitat suitability associated with warm and cold years, which may in part be because of diet differences, such as greater dependence on salmonids and squid [70].

Habitat selection in the basin was predominately driven by bottom temperature across all four complexes, with little contribution from other environmental variables at both the North and South complexes on St. George Island. Surface temperature and average phytoplankton biomass were more important for the two St. Paul complexes, although results from English Bay should be considered preliminary since we had extremely limited basin data for this complex. Fur seals from the North and South complexes had more distinct associations with bottom temperature, selecting against cold areas (< 2 °C) and for areas between 2 and 5 °C. This is likely due to association with slope habitat that is influenced by the Bering Slope Current and part of the Bering Sea “greenbelt”, a highly productive area that attracts large numbers of marine predators [96, 97]. While fur seals from Reef Point and English Bay also used slope habitat, habitat suitability was more diffuse and largely excluded the area around the Bering Canyon where suitable habitat for the North and South complexes was concentrated. Suitable habitat for all four complexes was present in more oceanic waters away from the slope, which likely reflects their use of mesoscale eddies in these regions [35]. Association with transient eddies would explain the reduced model performance of the Reef Point model given the challenges of adequately capturing the real-world locations of these features in the ROMS-NPZ model.

### Fisheries overlap

There was high overlap between core habitat of northern fur seals and commercial fishery catches of pollock during the “B” season. Spatial overlap alone does not necessarily imply there is competition between the fishery and fur seals, but there is overwhelming support that juvenile and adult pollock have been an important prey resource for fur seals in the eastern Bering Sea for over a century, particularly those breeding on St. Paul Island [32, 98–101]. For example, between 1995 and 2010 pollock comprised an estimated 41.4–76.5% of the prey biomass consumed by Pribilof Island fur seals [32]. There was considerable interannual variation in the age class of pollock consumed, with juvenile pollock comprising 16.6 − 63.2% of pollock biomass consumption, indicating availability of mature pollock is likely important in some years. In a community-wide survey, over 50% of Pribilof Island community members responded that nutritional limitation was the reason for the ongoing fur seal decline, with some responses indicating concern about how commercial fisheries are affecting food availability [33]. The total average annual “B” season fishery removals in the eastern Bering Sea from 2000 to 2022 were 750,968 t, with a range of 481,264 t − 893,162 t (see Table 1 in [102]). Assuming the catch data used in the analysis are representative of the entire “B” season catch, an average of 570,736 t of pollock are removed annually by the fishery from core habitat of lactating females within their foraging range (the 375 km circle). We did not consider fishery removals during the “A” season (January – April) because there is no temporal overlap with lactating fur seals during this time, although catches generally occur in identified areas of core habitat [102]. Our results further underscore the need for targeted studies investigating overlap and potential fisheries impacts on fur seals in a changing climate, which may need to occur at the complex level given current and projected variation in core habitat use.

### Projected distributions

There were considerable changes in core habitat characteristics under projected environmental conditions, but the magnitude and spatial predictions of core habitat availability varied considerably even among complexes with similar hindcasted distributions. While projected distributions provide an important first step in understanding how future environmental changes could impact this population, there are limitations associated with doing so, including uncertainty in future conditions and the assumption that identified relationships with environmental variables will remain unchanged in the future. The latter assumption is almost certainly to be violated because physical and biological conditions around the Pribilof Islands are changing, and as central-place foragers, lactating female fur seals have a limited ability to alter how far they travel from terrestrial breeding sites. Despite this, projections based on current habitat selection are still useful because they provide insight into the magnitude of change that fur seals are likely to experience and help identify a range of potential responses that can inform research efforts and ecosystem-based management. For example, the relatively large changes in projected habitat suitability for some complexes indicate that in the coming decades there may be shifts in at-sea spatial overlap among complexes, or potentially a redistribution among terrestrial sites as animals select for areas closer to more suitable foraging habitat. They also suggest that complexes may be differentially affected by future conditions, which could further exacerbate existing complex- and island-specific differences in population dynamics or lead to new ones. As suitable habitat is not necessarily optimal habitat, understanding how hindcasted habitat suitability translates to reproductive success and demographic trends will be key in identifying the potential ramifications of projected changes on fur seal population dynamics.

## Conclusions

Central-place marine foragers are constrained in their habitat use given their need to return to terrestrial sites for resting and reproduction. The distribution of available habitat is particularly important when this constraint occurs during reproduction given longer parental absences can adversely affect offspring growth and survival. Our study highlights how fine-scale spatial distribution in terrestrial sites influences marine habitat selection of lactating female northern fur seals, which in turn results in site-specific variation in projected changes in habitat suitability under future conditions. While our findings are perhaps not surprising given spatial variation in movements were already documented for this population, such comprehensive spatial coverage is atypical of many populations of marine central-place foragers. Even for fur seals on the Pribilof Islands, spatial segregation in habitat selection likely occurs at a finer spatial scale than examined here [48]. Our results underscore the need to adequately sample multiple sites whenever possible and carefully consider spatial scale when extrapolating model output for central-place foragers. For northern fur seals, this study is a key step towards providing data for climate-informed species and ecosystem-based fisheries management. Further efforts to improve upon the model described here, both from incorporating new fur seal movement data and advancements in ocean modeling simulations, remains critical for model improvement in projecting habitat suitability in an environment that will become increasingly novel in the coming decades.

## Electronic supplementary material

Below is the link to the electronic supplementary material.

Supplementary Material 1

## Data Availability

ROMS source code can be found on GitHub (https://github.com/beringnpz/roms-bering-sea). The specific release of the code used for the ACLIM Phase 2 simulations is archived at Zenodo (10.5281/zenodo.7062782). ACLIM simulation output is publicly available at https://data.pmel.noaa.gov/aclim/thredds/catalog/files.html. The data that support the findings of this study are publicly available at https://doi.org/10.5061/dryad.d51c5b0cd.
