# Supplementary material for: Current and future habitat suitability of northern fur seals and overlap with the commercial walleye pollock fishery in the eastern Bering Sea
Source: Mov Ecol. 2025 Apr 14;13:26. doi: 10.1186/s40462-025-00545-6 (PMC11995594; doi:10.1186/s40462-025-00545-6)
Supplement: Supplementary file 1 — Supplementary Material 1 [file 40462_2025_545_MOESM1_ESM.docx]

**Additional Material 1**

**Current and future habitat suitability of northern fur seals and overlap with the commercial walleye pollock fishery in the eastern Bering Sea**

**1. Results**

**
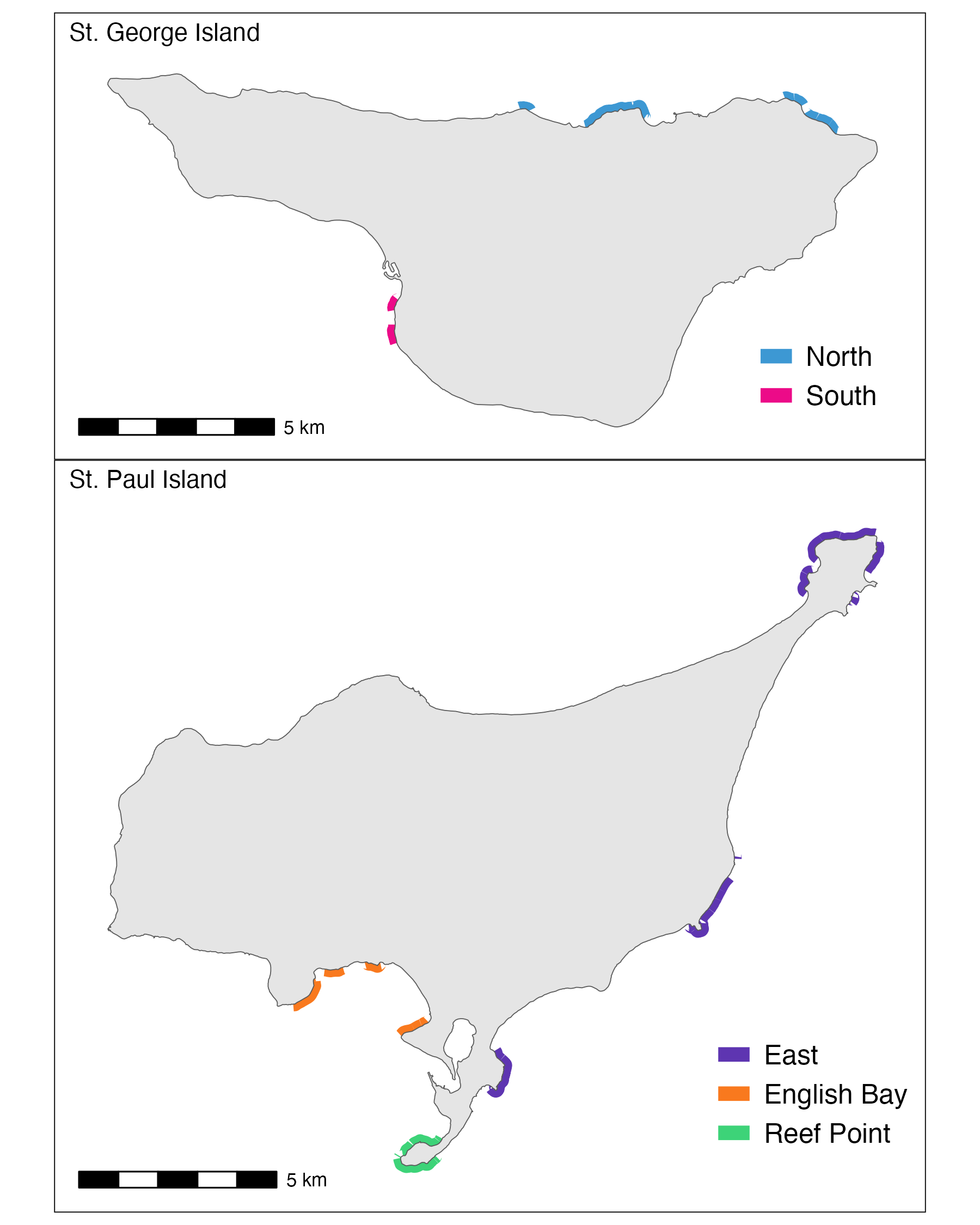
**

Figure S1. Terrestrial locations associated with the two rookery complexes on St. George Island and the three complexes on St. Paul Island.


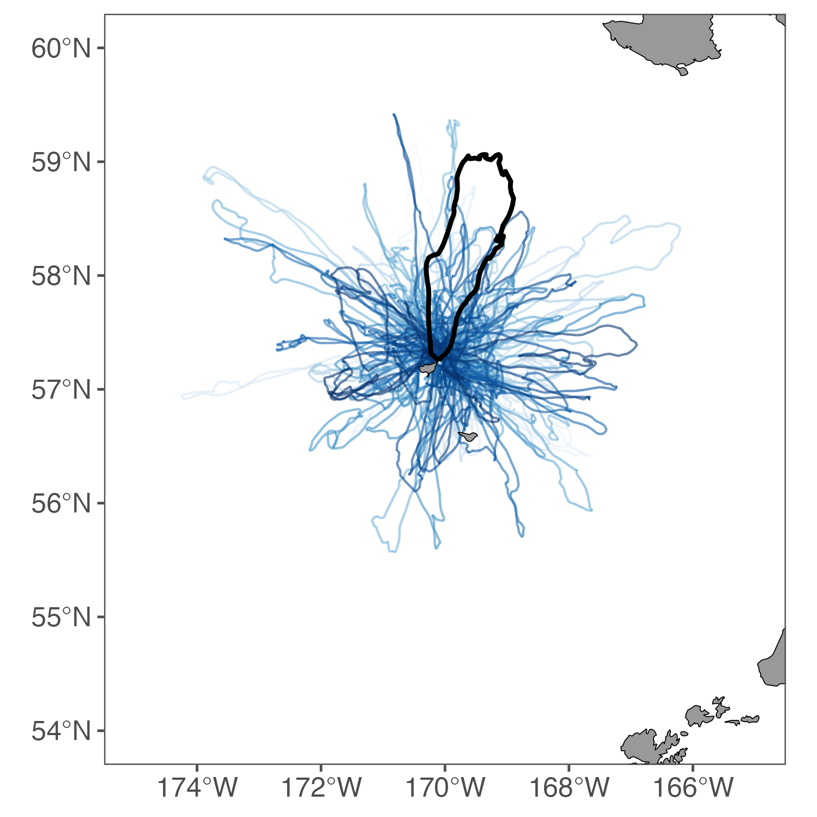


Figure S2. Example of 100 simulated tracks generated for a single foraging trip by an adult female northern fur seal instrumented at St. Paul Island. The actual trip is shown in black while simulated tracks are shown in shades of blue.


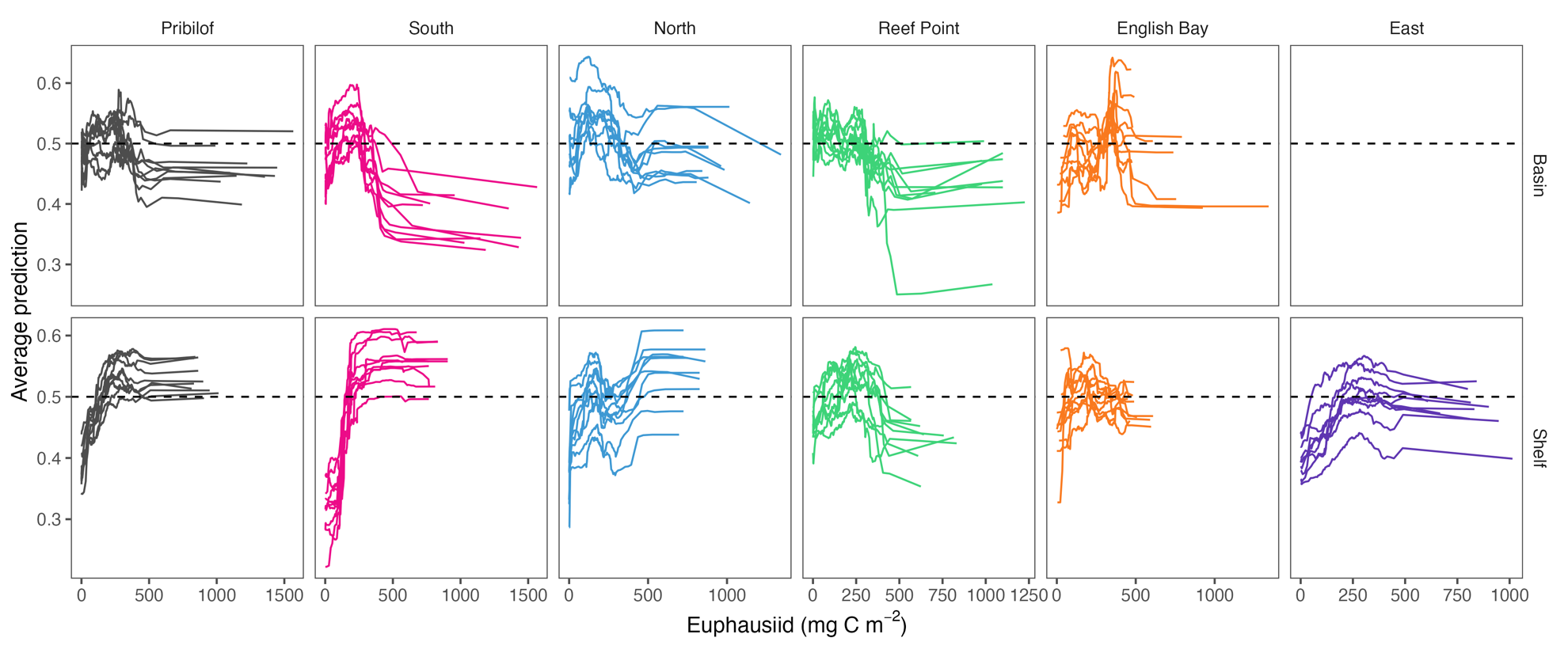


Figure S3. Partial dependence plots for euphausiid biomass from northern fur seal habitat selection models, separated by model (Pribilof wide and individual complex-specific models) and habitat type (basin vs. continental shelf).


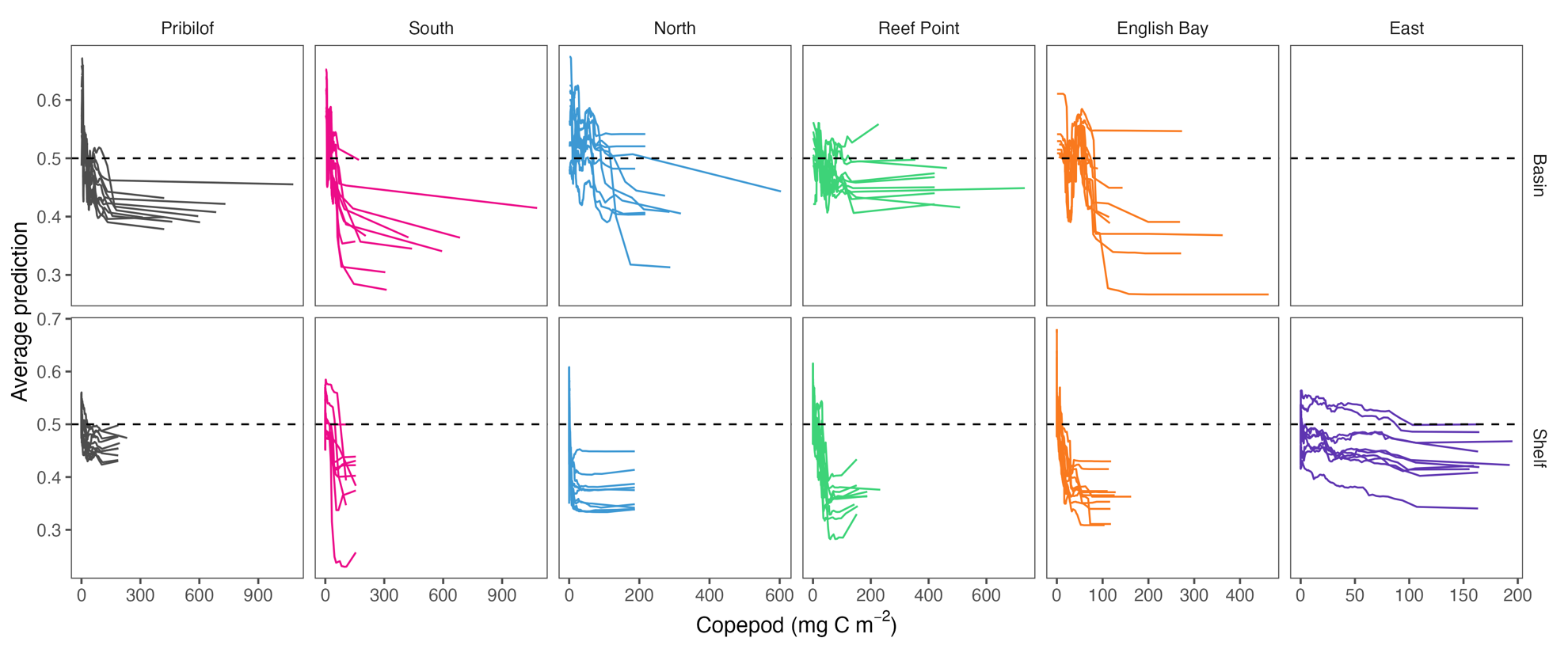


Figure S4. Partial dependence plots for copepod biomass from northern fur seal habitat selection models, separated by model (Pribilof wide and individual complex-specific models) and habitat type (basin vs. continental shelf).


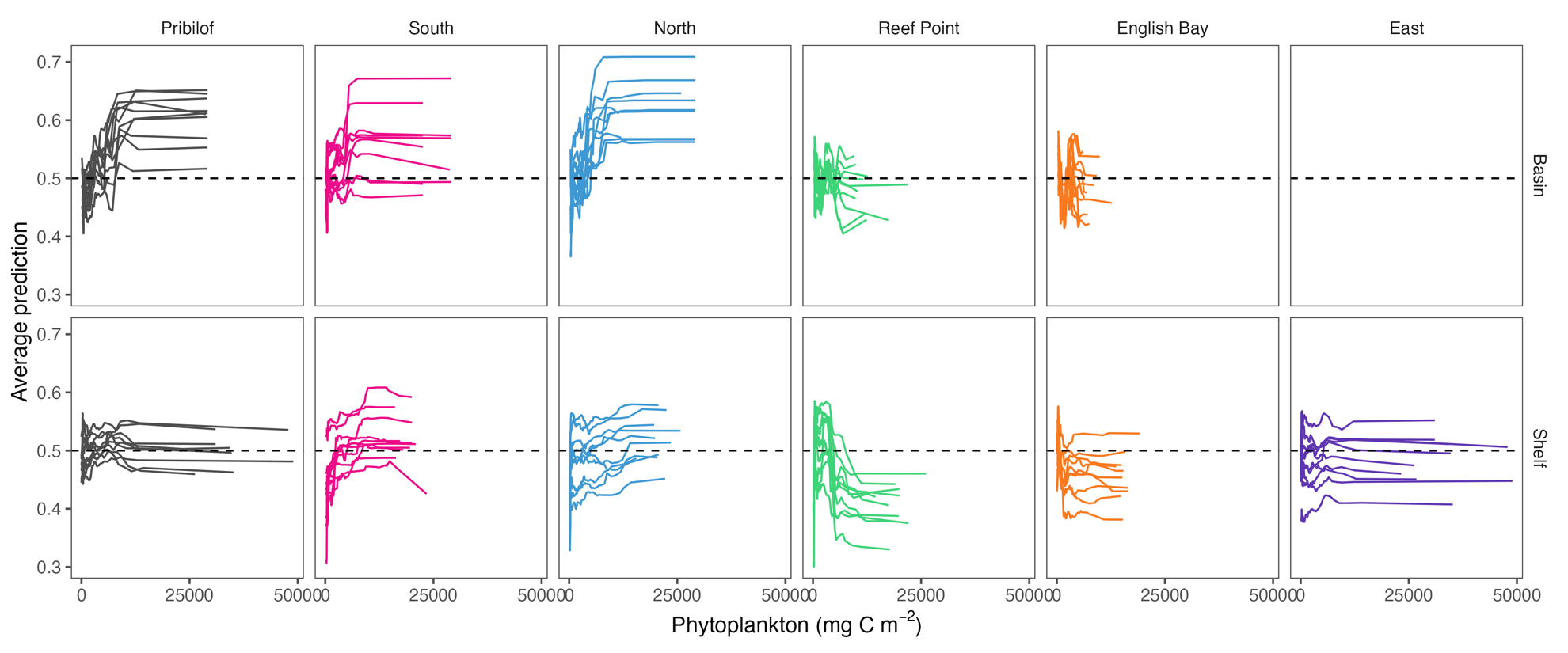


Figure S5. Partial dependence plots for phytoplankton biomass from northern fur seal habitat selection models, separated by model (Pribilof wide and individual complex-specific models) and habitat type (basin vs. continental shelf).


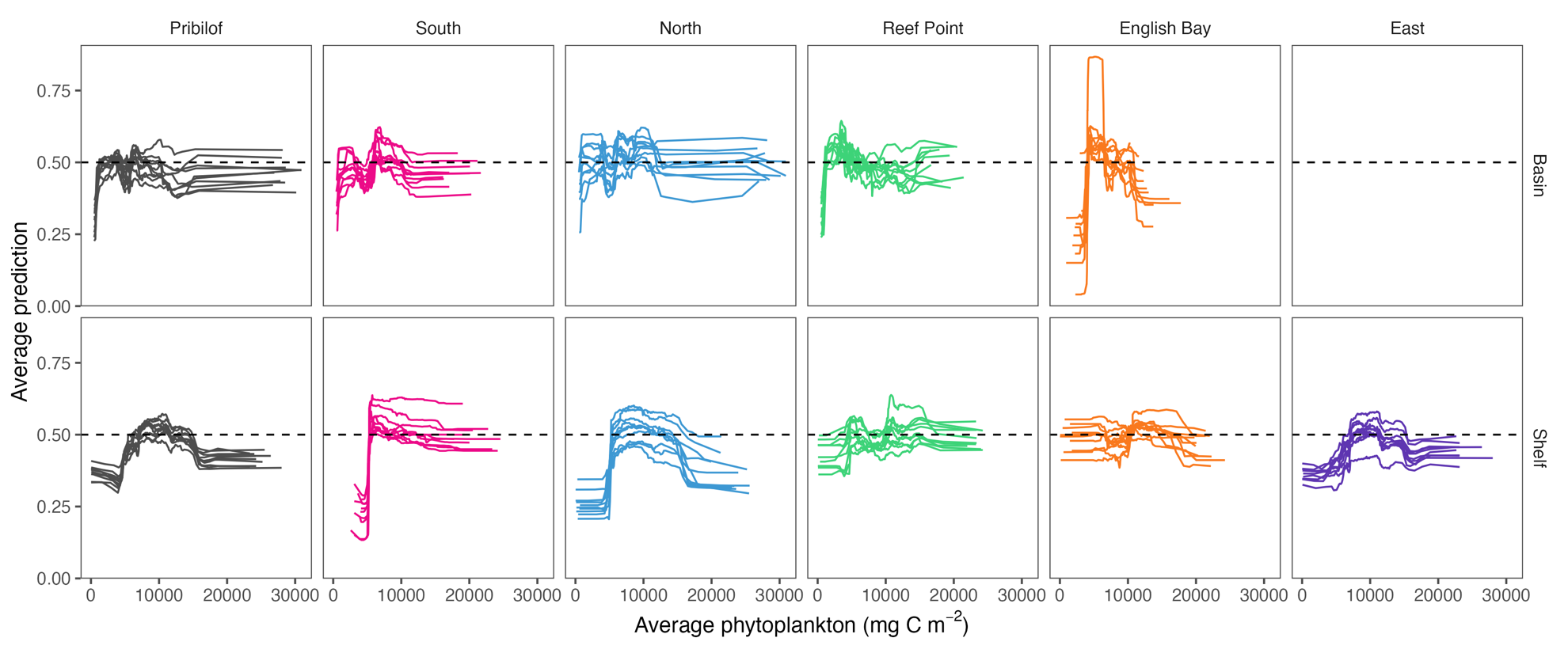


Figure S6. Partial dependence plots for average phytoplankton biomass from northern fur seal habitat selection models, separated by model (Pribilof wide and individual complex-specific models) and habitat type (basin vs. continental shelf).


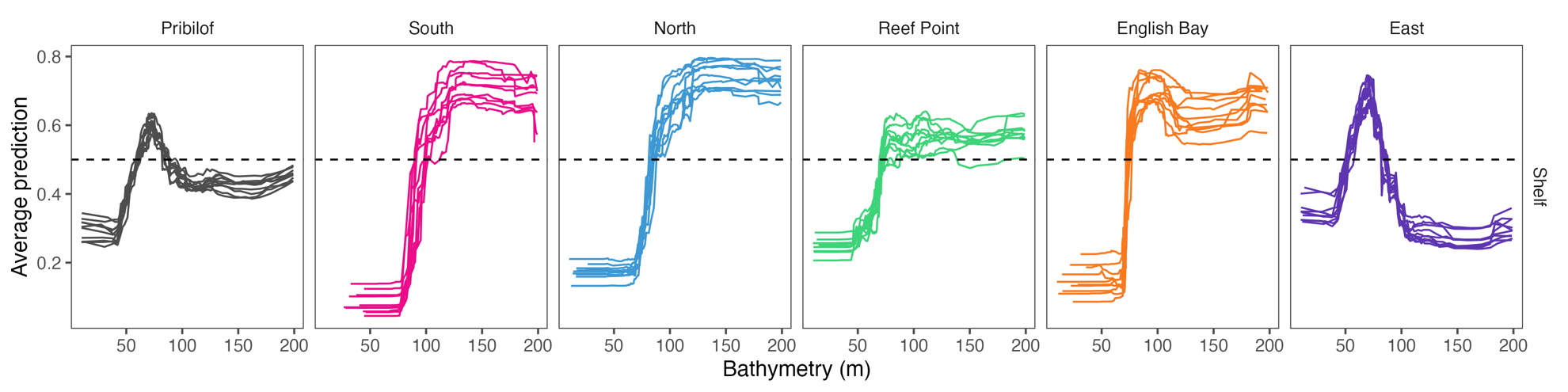


Figure S7. Partial dependence plots for bathymetry from northern fur seal habitat selection models, separated by model (Pribilof wide and individual complex-specific models).


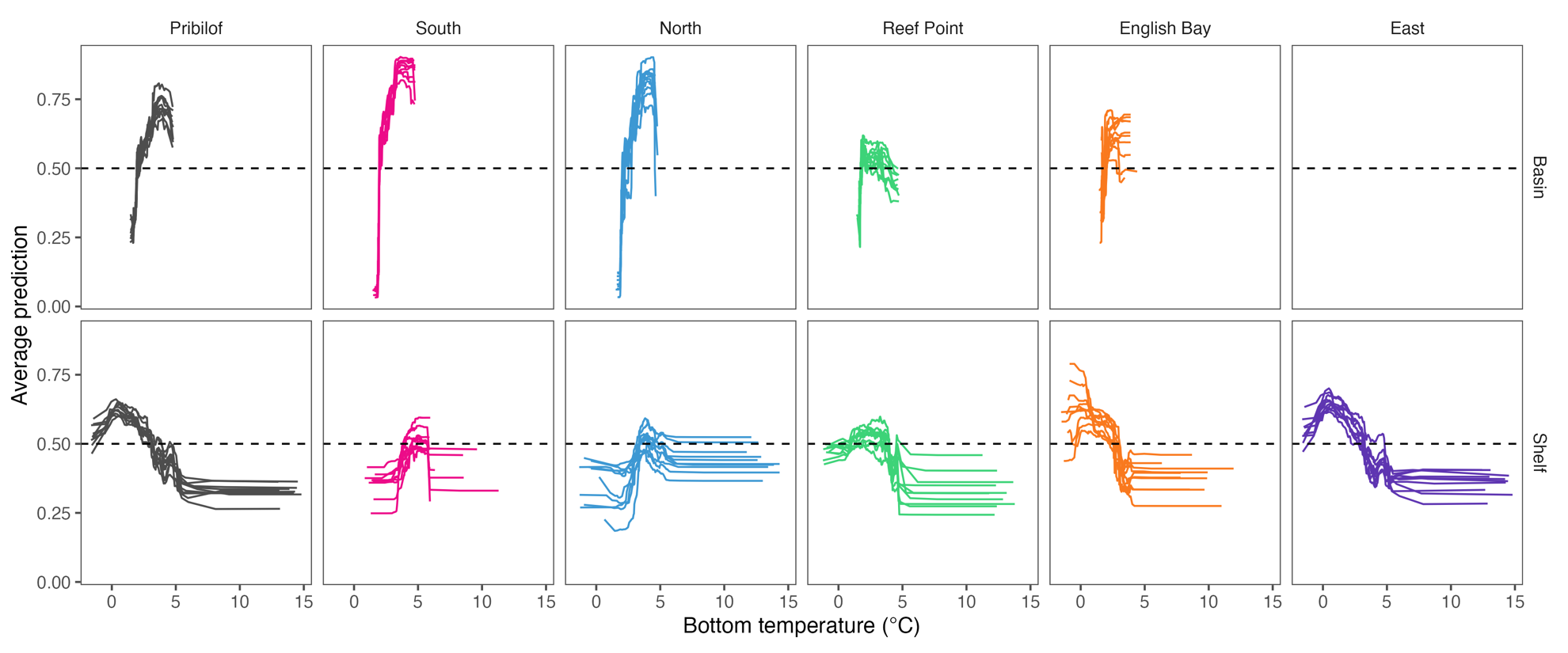


Figure S8. Partial dependence plots for bottom temperature from northern fur seal habitat selection models, separated by model (Pribilof wide and individual complex-specific models) and habitat type (basin vs. continental shelf).


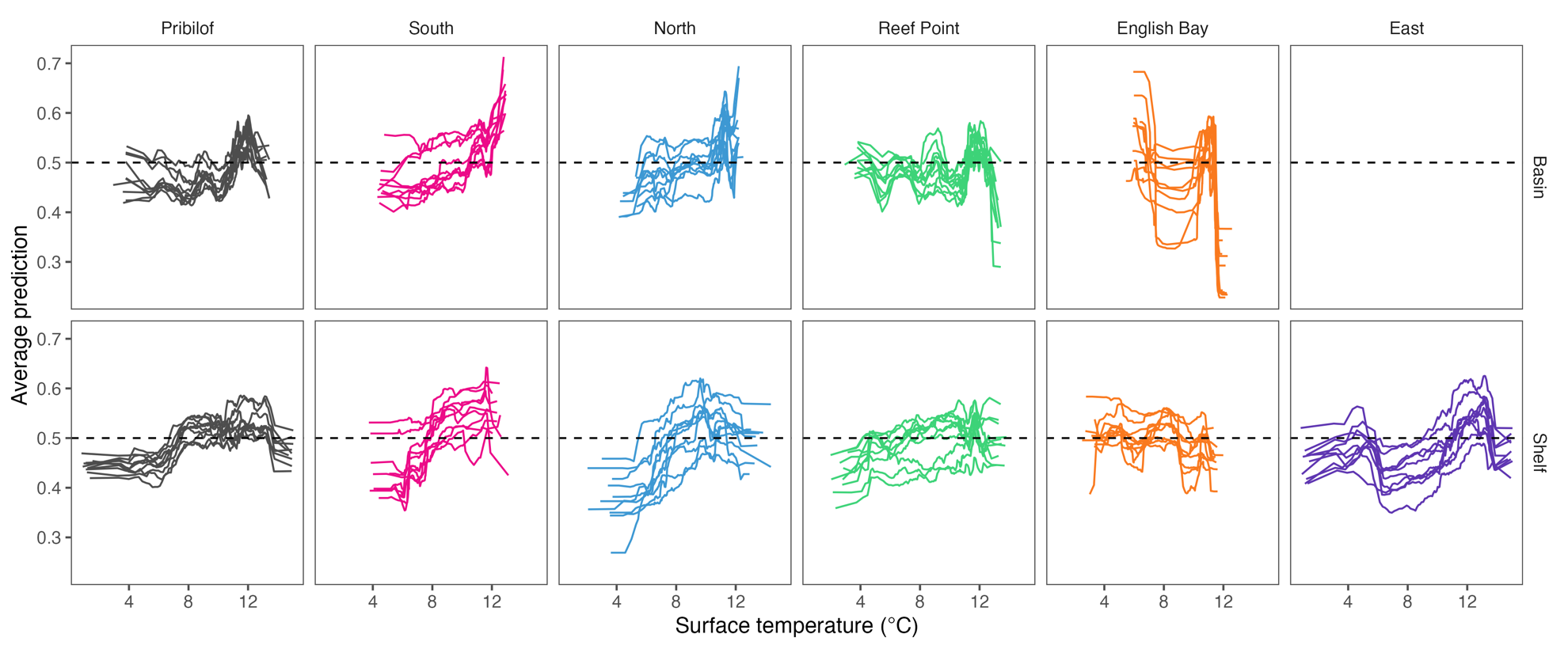


Figure S9. Partial dependence plots for surface temperature from northern fur seal habitat selection models, separated by model (Pribilof wide and individual complex-specific models) and habitat type (basin vs. continental shelf).


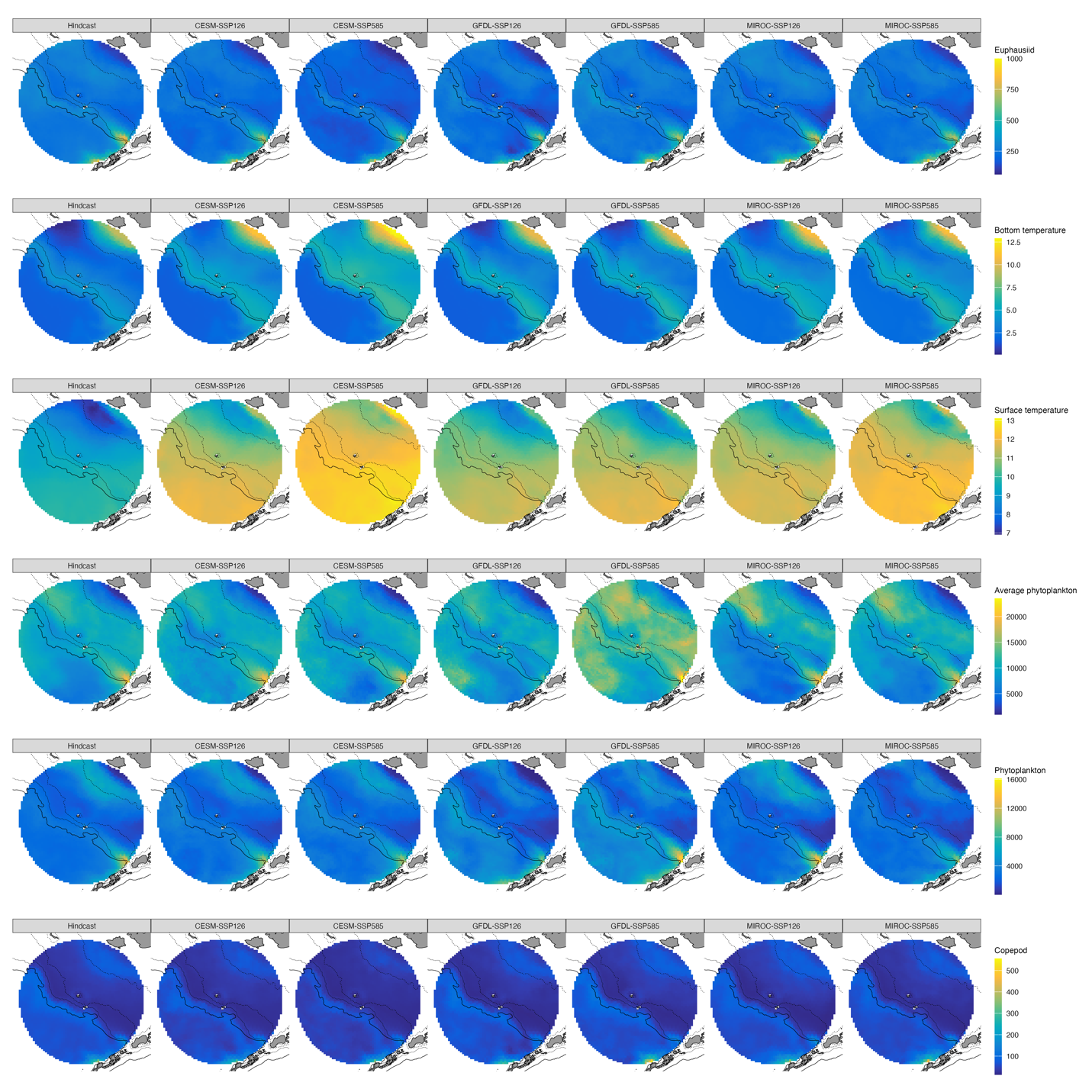


Figure S10. Average environmental conditions between July and October from the ROMS-NPZ model for the hindcast (left, 1992 – 2018) and each Earth System Model-shared socioeconomic pathway (2050 – 2059). In each subplot the solid black line represents the 200 m isobath, while the dashed lines represent the ROMS-NPZ isobaths (200 m, 100 m, and 50 m, from left to right). Units are as follows: mg C m^-2^ (euphausiid, phytoplankton, copepod), m (bathymetry), and ℃ (bottom and surface temperature).


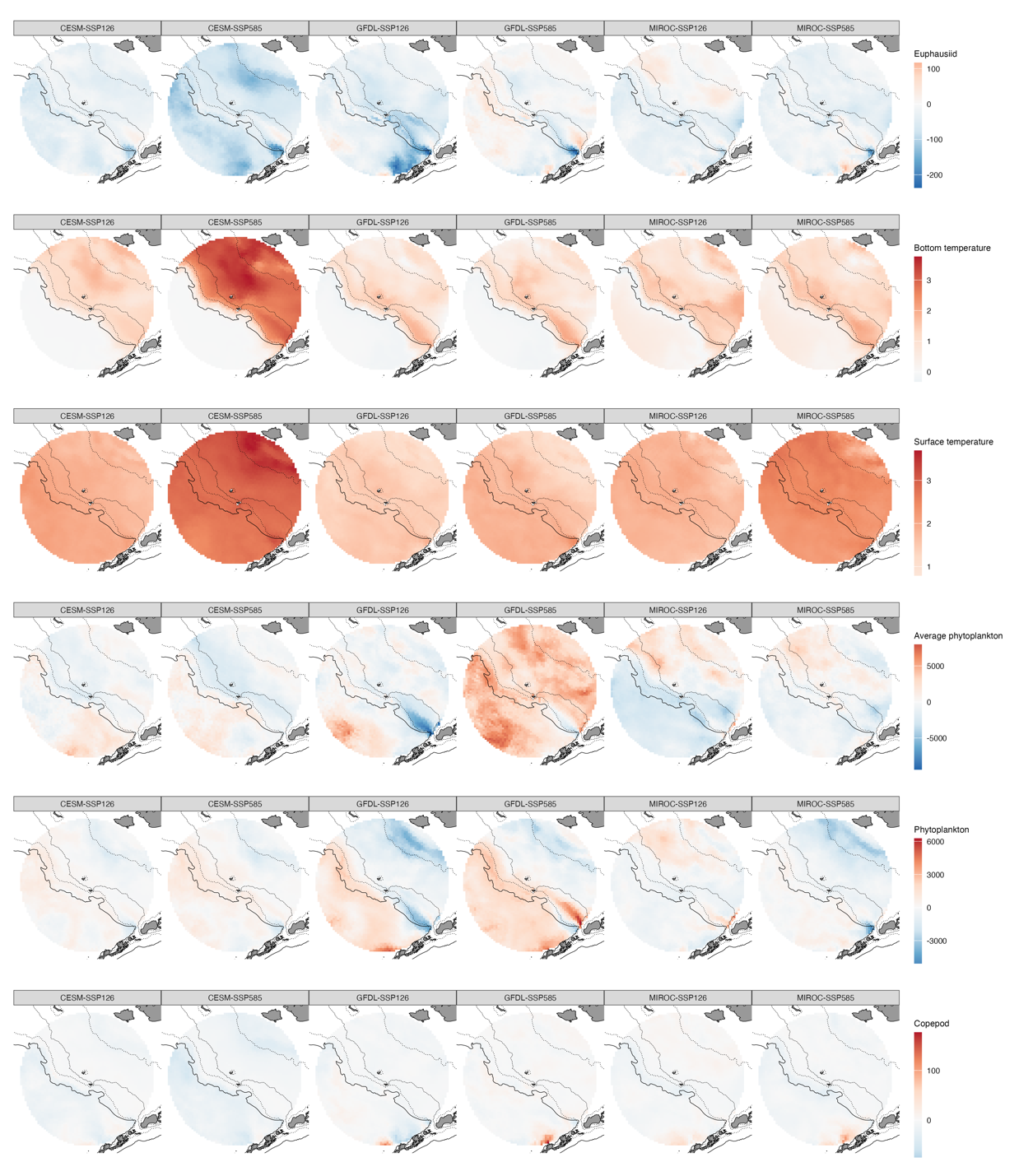


Figure S11. Difference in average environmental conditions between July and October from the ROMS-NPZ model for the hindcast (1992 – 2018) and each Earth System Model-shared socioeconomic pathway (2050 – 2059). Warm colors indicate projected average environmental conditions are higher than the hindcast and cooler colors the opposite. In each subplot the solid black line represents the 200 m isobath, while the dashed lines represent the ROMS-NPZ isobaths (200 m, 100 m, and 50 m, from left to right). Units are as follows: mg C m^-2^ (euphausiid, phytoplankton, copepod), m (bathymetry), and ℃ (bottom and surface temperature).


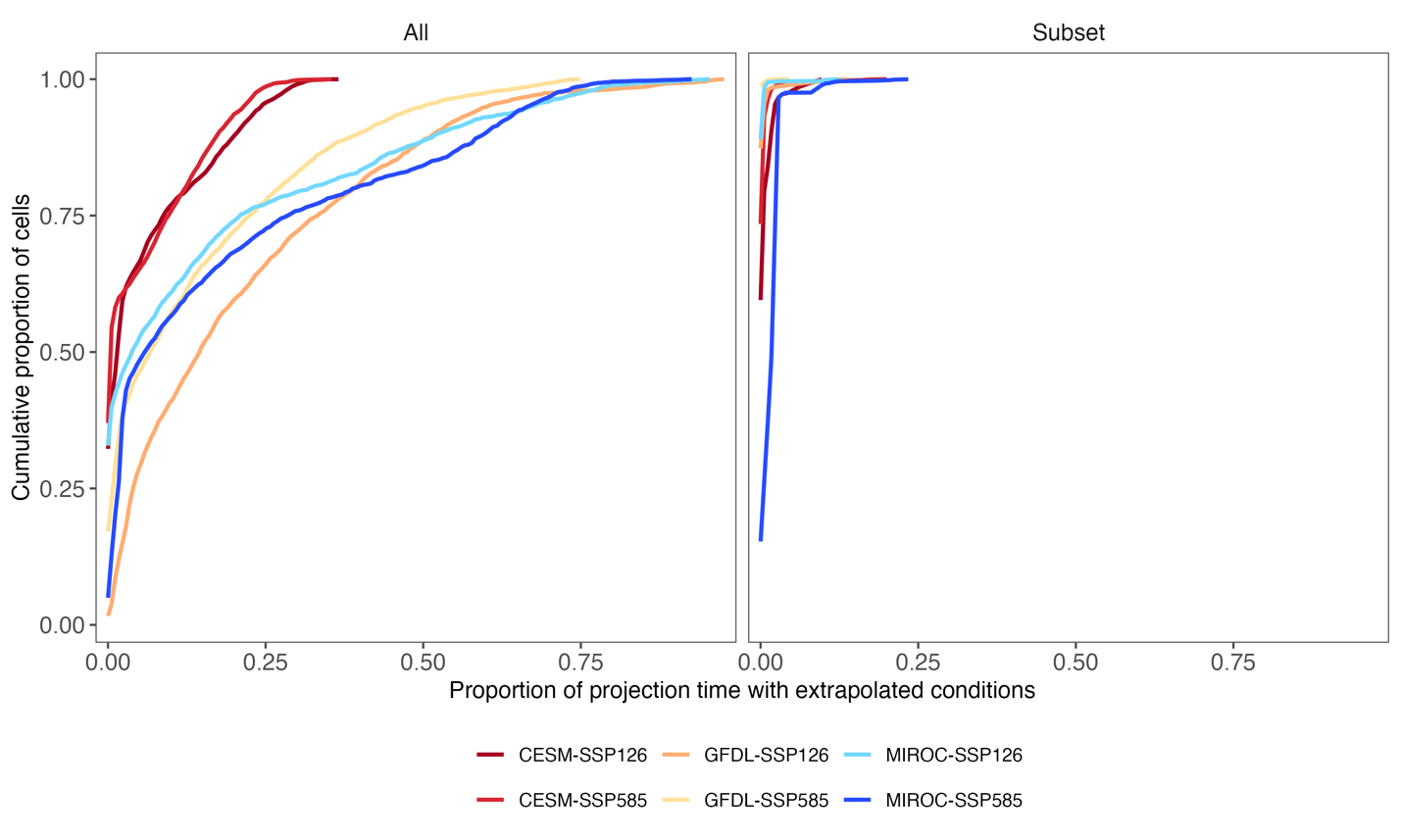


Figure S12. The cumulative proportion of cells within the projection area with extrapolated conditions as a function of the proportion of the projection interval for all variables (All) or excluding three biological variables (large phytoplankton, copepod, and euphausiid biomass) as described in text (Subset). Colors indicate the different Earth System Model - shared socioeconomic pathway (SSP) combinations. The plots highlight that relatively few cells had extrapolated conditions, where one or more variables was outside of the reference range, and that even when present, extrapolated conditions only occurred for a short amount of the projection period.


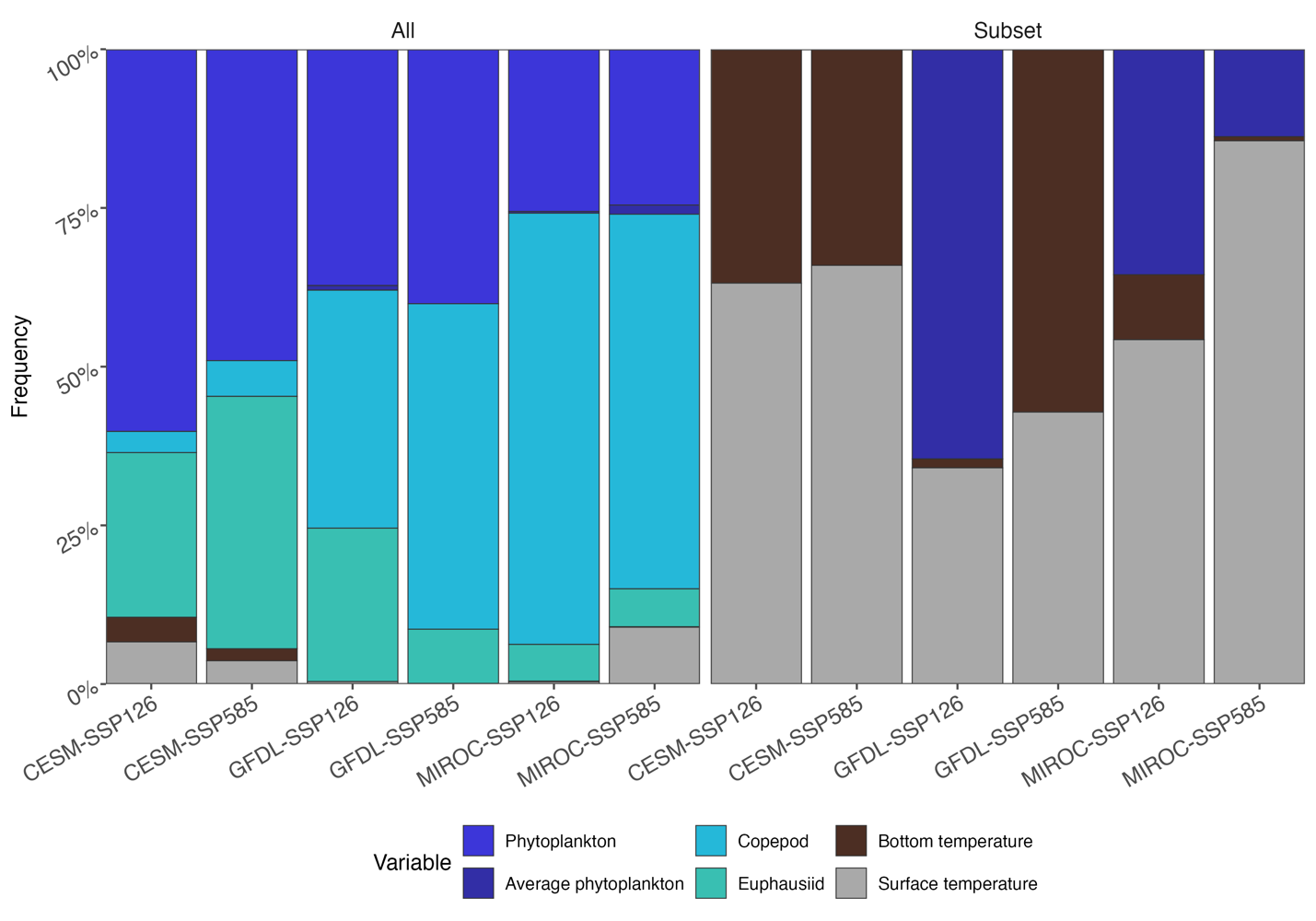


Figure S13. Contribution of each ROMS-NPZ variable to extrapolated conditions during the projection period (2050 – 2059) for each Earth System Model - shared socioeconomic pathway (SSP) combination. The left subplot shows results when all variables were included (All), whereas the right subplot shows the contribution excluding three biological variables (large phytoplankton, copepod, and euphausiid biomass) as described in text (Subset).


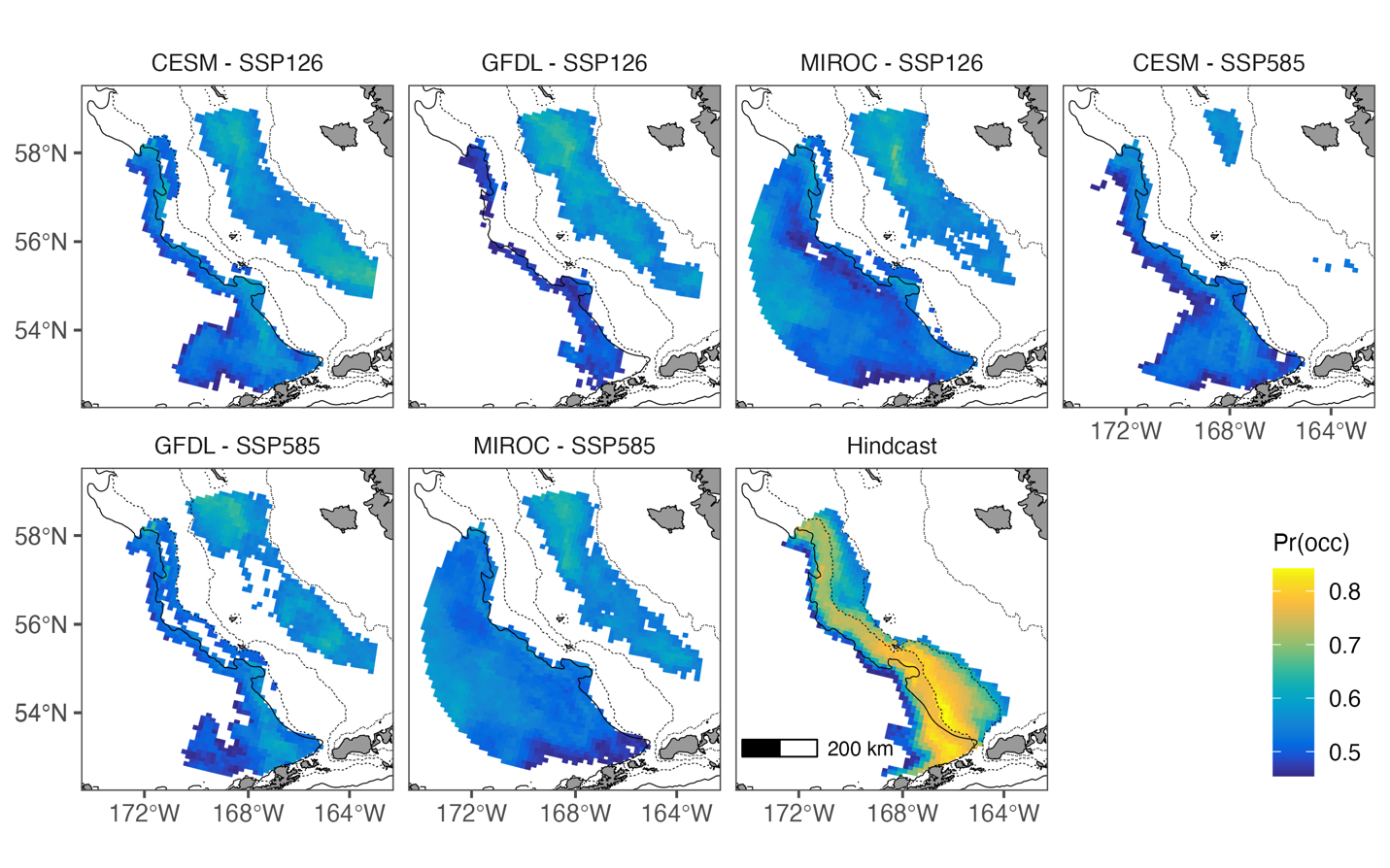


Figure S14. Spatial predictions of mean core habitat suitability for lactating adult female northern fur seals from the North complex under projection conditions (2050 – 2059). Predictions are shown separately for each Earth System Model - shared socioeconomic pathway (SSP) combination. Hindcasted (1992 – 2018) mean core habitat suitability is shown for reference. The solid black line represents the 200 m isobath, whereas the dotted lines represent the ROMS-NPZ 200 m, 100m, and 50 m isobaths (from left to right). Predictions between the actual and ROMZ-NPZ 200 m isobath represent average predictions from shelf and basin models. The scale bar is the same across all subplots


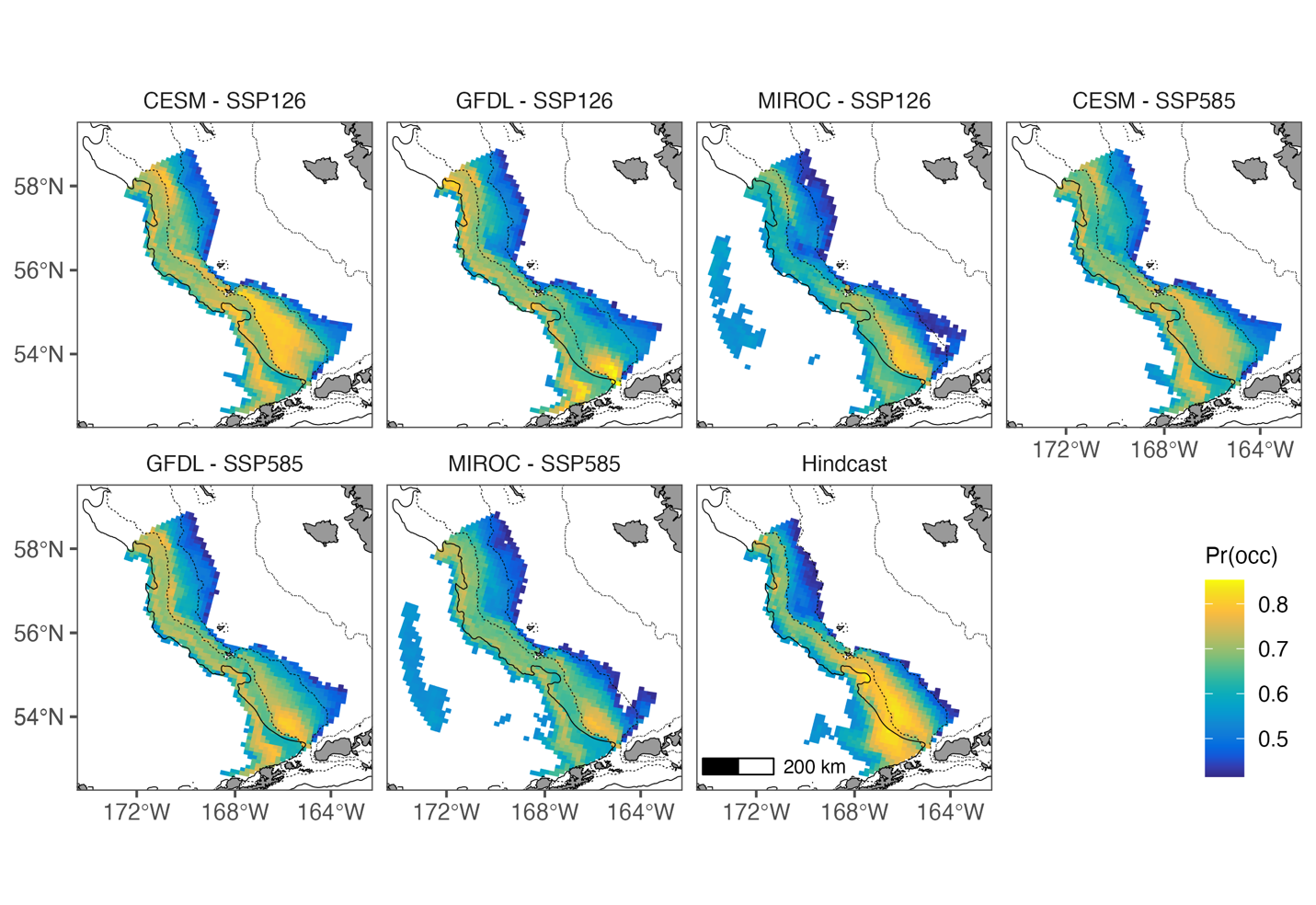


Figure S15. Spatial predictions of mean core habitat suitability for lactating adult female northern fur seals from the South complex under projection conditions (2050 – 2059). Predictions are shown separately for each Earth System Model - shared socioeconomic pathway (SSP) combination. Hindcasted (1992 – 2018) mean core habitat suitability is shown for reference. The solid black line represents the 200 m isobath, whereas the dotted lines represent the ROMS-NPZ 200 m, 100m, and 50 m isobaths (from left to right). Predictions between the actual and ROMZ-NPZ 200 m isobath represent average predictions from shelf and basin models. The scale bar is the same across all subplots


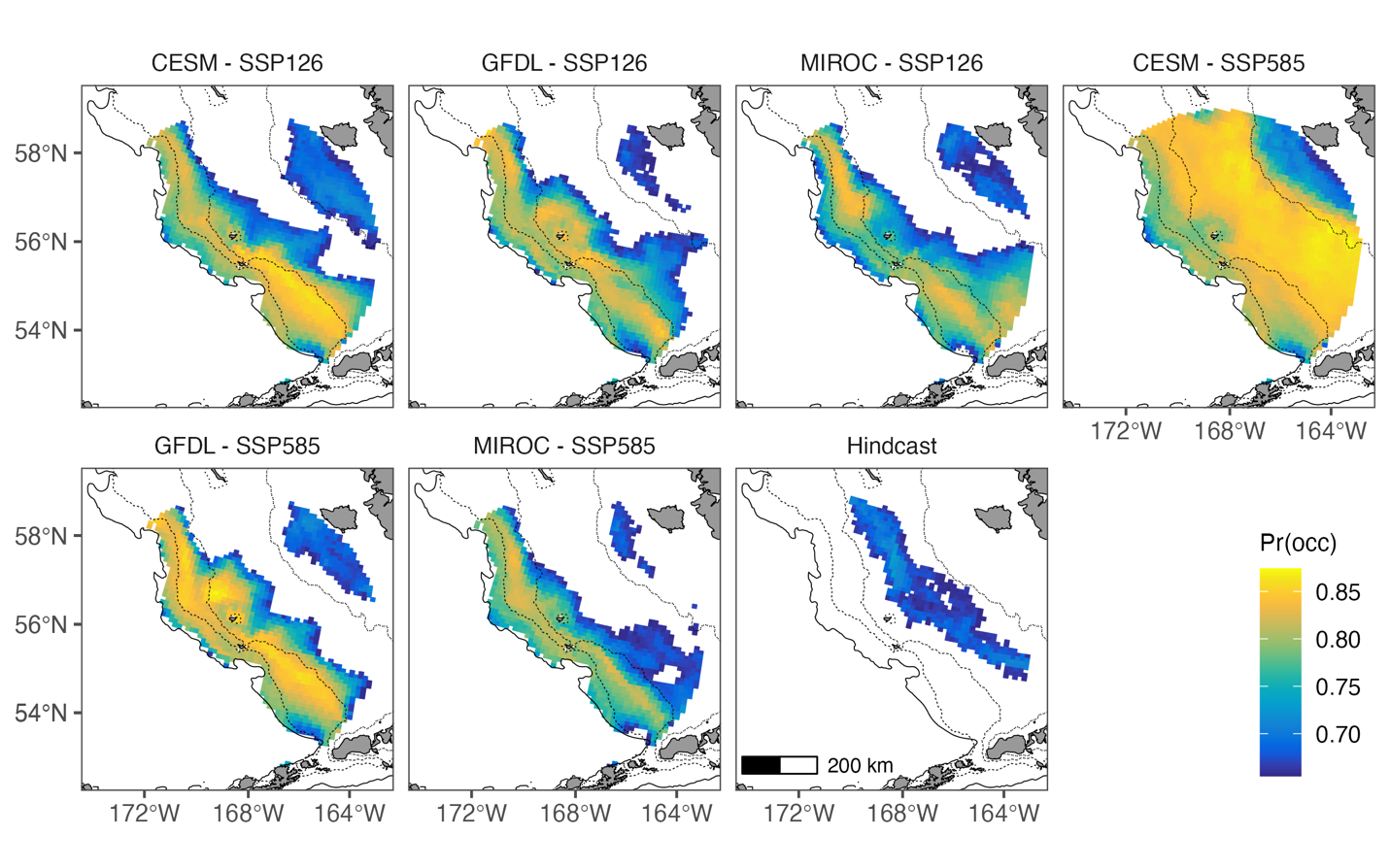


Figure S16. Spatial predictions of mean core habitat suitability for lactating adult female northern fur seals from the East complex under projection conditions (2050 – 2059). Predictions are shown separately for each Earth System Model - shared socioeconomic pathway (SSP) combination. Hindcasted (1992 – 2018) mean core habitat suitability is shown for reference. The solid black line represents the 200 m isobath, whereas the dotted lines represent the ROMS-NPZ 200 m, 100m, and 50 m isobaths (from left to right). The scale bar is the same across all subplots.


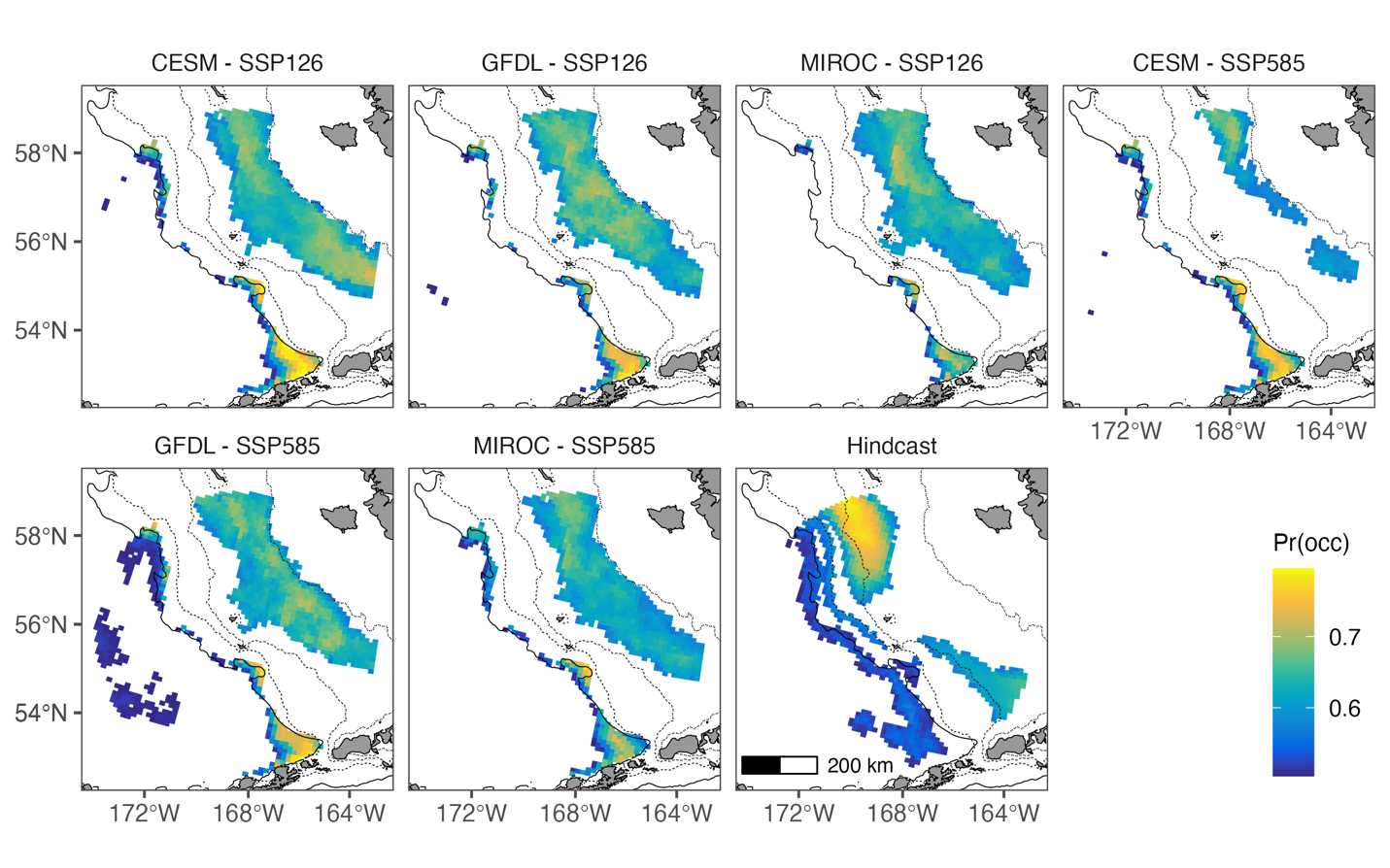


Figure S17. Spatial predictions of mean habitat suitability for lactating adult female northern fur seals from the St. Paul - English Bay complex under projection conditions (2050 – 2059). Predictions are shown separately for each Earth System Model - shared socioeconomic pathway (SSP) combination. Hindcasted (1992 – 2018) mean habitat suitability is shown for reference. The solid black line represents the 200 m isobath, whereas the dotted lines represent the ROMS-NPZ 200 m, 100m, and 50 m isobaths (from left to right). Predictions between the actual and ROMZ-NPZ 200 m isobath represent average predictions from shelf and basin models. The scale bar is the same across all subplots.


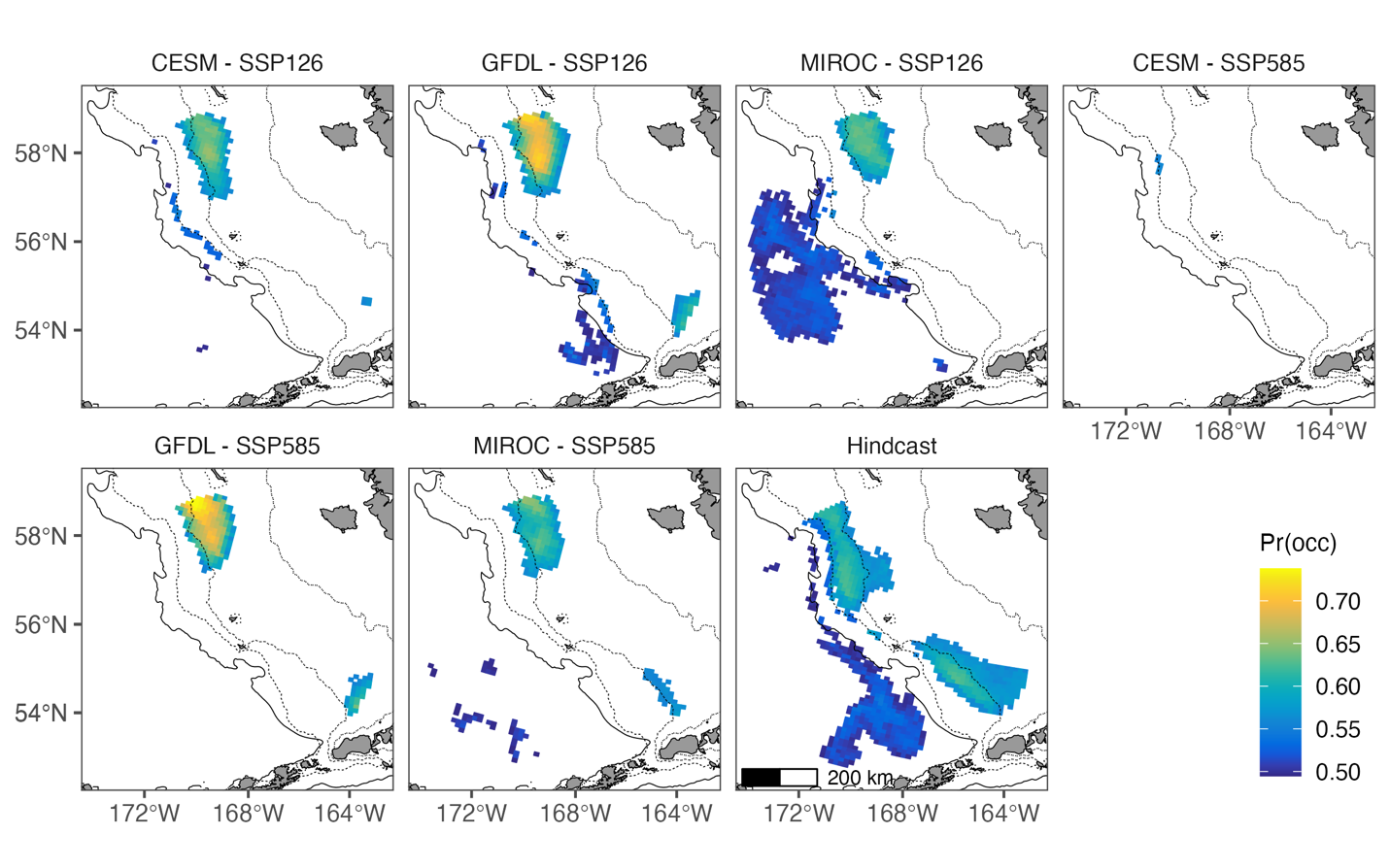


Figure S18. Spatial predictions of mean core habitat suitability for lactating adult female northern fur seals from the Reef Point complex under projection conditions (2050 – 2059). Predictions are shown separately for each Earth System Model - shared socioeconomic pathway (SSP) combination. Hindcasted (1992 – 2018) mean core habitat suitability is shown for reference. The solid black line represents the 200 m isobath, whereas the dotted lines represent the ROMS-NPZ 200 m, 100m, and 50 m isobaths (from left to right). Predictions between the actual and ROMZ-NPZ 200 m isobath represent average predictions from shelf and basin models. The scale bar is the same across all subplots.


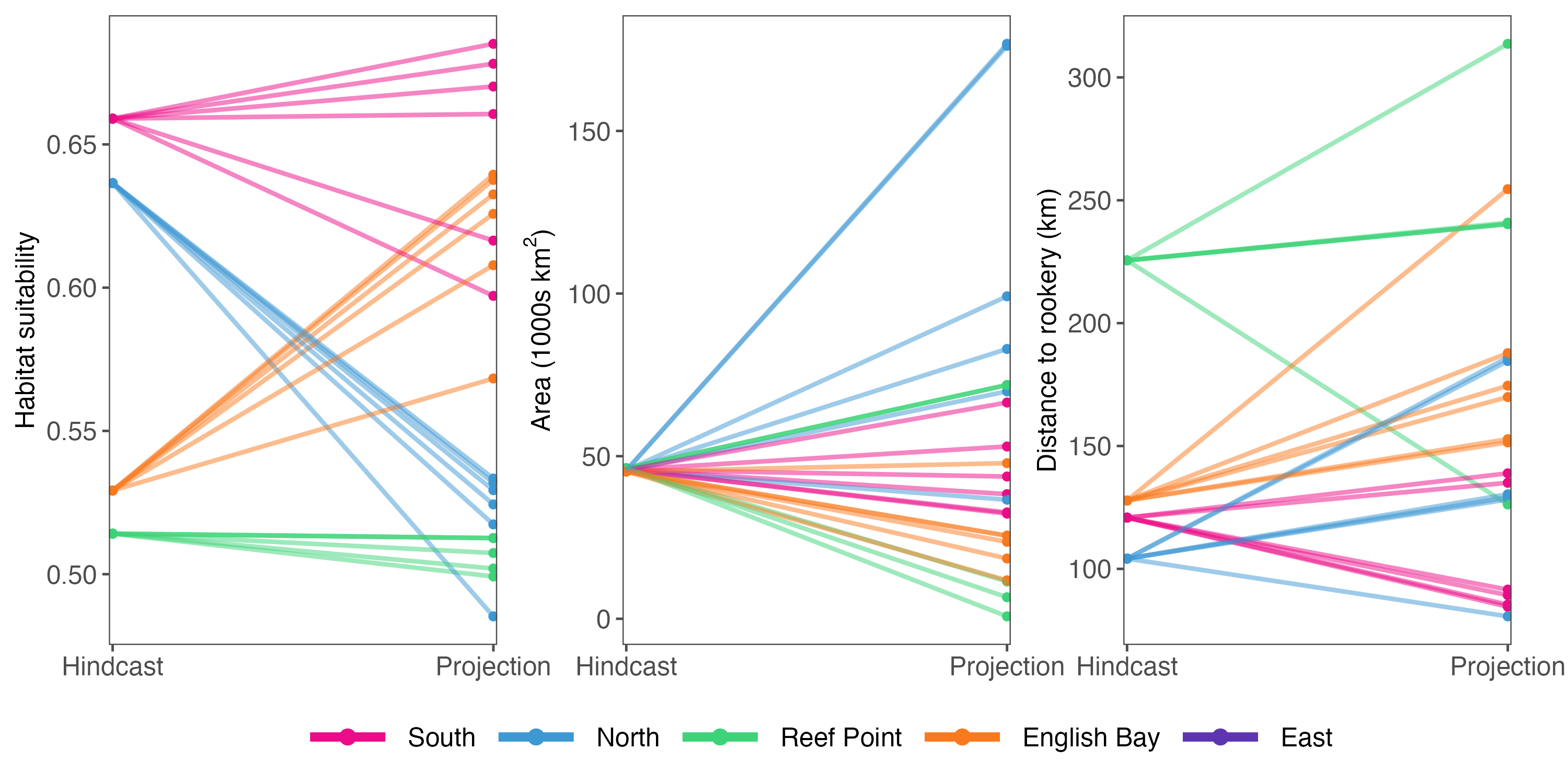


Figure S19. Changes in fur seal habitat suitability metrics in the basin between hindcasts (1992 – 2018) and projections (2050 – 2059). Metrics shown are the mean core habitat suitability, the total area of core habitat, and the mean distance between the center of gravity of core habitat and either St. Paul or St. George Island. Each projection point represents a single Earth System Model-shared socioeconomic pathway combination.

Table S1. Metrics of model performance for northern fur seal habitat models based on 10-fold cross validation, including the area under the receiver operating curve (AUC) and the Matthews correlation coefficient (MCC), as output by the function *evalm* (package “MLeval” v. 0.3; [70]. A threshold of 0.5 was used for relevant metrics where probabilities were converted to presence or absence. Values for each model represent averages across all replicate populations. See main text for description of individual model and assessment of performant.

| **Model** | **AUC** | **MCC** | **Precision** | **Sensitivity** | **Specificity** |
| --- | --- | --- | --- | --- | --- |
| *Continental shelf* |  |  |  |  |  |
| Pribilof | 0.88 | 0.58 | 0.78 | 0.81 | 0.77 |
| East | 0.91 | 0.65 | 0.79 | 0.87 | 0.77 |
| English Bay | 0.86 | 0.56 | 0.77 | 0.81 | 0.75 |
| Reef Point | 0.74 | 0.33 | 65 | 0.71 | 0.62 |
| North | 0.95 | 0.77 | 0.86 | 0.91 | 0.85 |
| South | 0.96 | 0.81 | 0.91 | 0.91 | 0.91 |
| *Basin* |  |  |  |  |  |
| Pribilof | 0.81 | 0.47 | 0.75 | 0.70 | 0.76 |
| English Bay | 0.74 | 0.36 | 0.68 | 0.69 | 0.68 |
| Reef Point | 0.66 | 0.22 | 0.61 | 0.61 | 0.61 |
| North | 0.89 | 0.61 | 0.81 | 0.80 | 0.81 |
| South | 0.94 | 0.71 | 0.84 | 0.88 | 0.83 |

**2. Bias correction**

The ROMS-NPZ projections were adjusted to remove bias following the equation:

$$x_{c,t}^{projbc}=x_{c,t}^{proj}+(\mu_{c,w(t)}^{hind}-\mu_{c,w(t)}^{hist})$$

where $x_{c,t}^{proj}$ is a forecast data value in grid cell *c* at time *t*, $u_{c,w(t)}^{hind}$ is the mean hindcast value in cell *c* and week $w(t)$ across the 1985 - 2014 reference period, $\mu_{c,w(t)}^{hist}$ is the mean value in grid cell *c* and week $w(t)$ across the 1985 - 2014 historical period of the projection simulation, and and $x_{c,t}^{projbc}$ is the resulting bias corrected-value in cell *c* at time *t*. Bias-corrected values of biological variables (large phytoplankton, copepod, and euphausiid) were not allowed to be negative, and were forced to 0 when this occurred. Average phytoplankton values were calculated based on bias-corrected large phytoplankton values.
